# Cortical glutamate and GABA are related to compulsive behaviour in individuals with obsessive compulsive disorder and healthy controls

**DOI:** 10.1038/s41467-023-38695-z

**Published:** 2023-06-27

**Authors:** Marjan Biria, Paula Banca, Máiréad P. Healy, Engin Keser, Stephen J. Sawiak, Christopher T. Rodgers, Catarina Rua, Ana Maria Frota Lisbôa Pereira de Souza, Aleya A. Marzuki, Akeem Sule, Karen D. Ersche, Trevor W. Robbins

**Affiliations:** 1 Department of Psychology, University of Cambridge, Cambridge, CB2 3EB UK; 2 Behavioural and Clinical Neuroscience Institute, University of Cambridge, Cambridge, CB2 3EB UK; 3 Department of Physiology, Development and Neuroscience, University of Cambridge, Cambridge, CB2 3EL UK; 4 Wolfson Brain Imaging Centre, Department of Clinical Neurosciences, University of Cambridge, Cambridge, CB2 0QQ UK; 5 Department of Clinical Neurosciences and Cambridge University Hospitals NHS Trust, University of Cambridge, Cambridge, UK; 6 Department of Psychology, School of Medical and Life Sciences, Sunway University, Petaling Jaya, Malaysia; 7 Department of Psychiatry, School of Clinical Medicine, University of Cambridge, Cambridge, UK; 8 Department of Systems Neuroscience, University Medical Center Hamburg-Eppendorf, Hamburg, Germany; 9 Department of Addictive Behaviour and Addiction Medicine, Central Institute of Mental Health, University of Heidelberg, Heidelberg, Germany

**Keywords:** Cognitive control, Decision, Obsessive compulsive disorder

## Abstract

There has been little analysis of neurochemical correlates of compulsive behaviour to illuminate its underlying neural mechanisms. We use 7-Tesla proton magnetic resonance spectroscopy (^1^H-MRS) to assess the balance of excitatory and inhibitory neurotransmission by measuring glutamate and GABA levels in anterior cingulate cortex (ACC) and supplementary motor area (SMA) of healthy volunteers and participants with Obsessive-Compulsive Disorder (OCD). Within the SMA, trait and clinical measures of compulsive behaviour are related to glutamate levels, whereas a behavioural index of habitual control correlates with the glutamate:GABA ratio. Participants with OCD also show the latter relationship in the ACC while exhibiting elevated glutamate and lower GABA levels in that region. This study highlights SMA mechanisms of habitual control relevant to compulsive behaviour, common to the healthy sub-clinical and OCD populations. The results also demonstrate additional involvement of anterior cingulate in the balance between goal-directed and habitual responding in OCD.

## Abstract (web summary)

The neurochemical basis of compulsive behaviour is not well understood. Here, the authors show that levels of glutamate and GABA in the supplementary motor area and anterior cingulate cortex relate to compulsive behaviour in healthy controls and individuals with OCD.

## Introduction

Compulsivity can be defined as perseverative behaviour with potentially maladaptive consequences. The construct of compulsivity – a transdiagnostic psychiatric trait – has no clear boundary between the healthy and pathological parts of the spectrum^1^. Individual differences in compulsive behaviour have been related to functioning of discrete fronto-striatal ‘loops’ in individuals with compulsive traits^2^ and in psychiatric patients with extreme levels of compulsive symptoms such as those with substance use disorders^3^ and obsessive-compulsive disorder (OCD)^4^. Changes in fronto-striatal function underpinning compulsive behaviour may be influenced by neurochemical dysregulation of cortical networks. There is considerable evidence of hyperactivity in OCD in certain cortical regions based on blood oxygenation level dependent (BOLD) neuroimaging^4–6^, which presumably is a consequence of changes in the excitatory/inhibitory balance in cortical networks resulting from changes in glutamate (Glu) and γ-amino butyric acid (GABA) neurotransmission^7^. Abnormally high levels of Glu within OCD have been suggested in animal models and by human genetic, pharmacological and neurochemical studies^7–9^. However, there have been inconsistent findings using proton magnetic resonance spectroscopy (^1^H-MRS) to directly measure regional levels of these neurotransmitters (see Brennan et al.^10^ and Biria et al.^11^ for review). For example, one 3-Tesla (3T) ^1^H-MRS study found evidence of reduced GABA in the medial prefrontal cortex of participants with OCD, suggesting an altered excitatory/inhibitory balance in that region^12^; however, a later study found the reverse^13^.

Evidence for neurochemical dysregulation mediating compulsive behaviour has been hindered by a lack of high resolution quantification of Glu and its metabolite glutamine (Gln), as well as GABA using ^1^H-MRS at field strengths of 3T or lower. To overcome this limitation, we utilised 7-Tesla ^1^H-MRS and an optimised MRS sequence (semi-LASER) to reliably and separately quantify Glu, Gln and GABA in individuals with and without OCD. This enabled us to define more accurately a proxy neurochemical index of the balance between excitatory and inhibitory neurotransmission within the anterior cingulate cortex (ACC) and the supplementary motor area (SMA). The latter are key regions previously linked to compulsivity and strongly implicated in the pathophysiology of OCD. Since the vast majority of studies have found OCD brain dysfunction within the anterior cortex, we used the occipital cortex (OCC) as a posterior cortical comparison region.

The ACC is implicated in error monitoring^14,15^ and reward prediction errors^16,17^, which are cognitive processes critical for compulsive responses. Moreover, enhanced prediction errors and aberrant activity of the ACC are reported in OCD^18–20^. The SMA has also been implicated in error processing in OCD^21^ and participates in a sensorimotor circuit with the putamen^22^, also crucial for habit learning^23–25^. The neurocognitive deficits found in the SMA are considered an endophenotype of OCD, possibly related to inefficient neural processing, as measured during a response inhibition task^6^. The relevance of SMA in OCD is also apparent from symptom improvements following brain stimulation^26,27^.

We further correlated neurochemical levels within these regions with measures of compulsivity, and a behavioural index of habitual control- a contingency degradation task, whereby the association between an action and an outcome is uncoupled or degraded^23^. Since compulsive behaviour in OCD has been postulated to emerge from an imbalanced cortico-striatal circuitry favouring the habit system we hypothesised that this might be reflected in neurochemical imbalances in the anterior cingulate and SMA.

In summary we hypothesised that changes in the neurochemical properties of two key regions of fronto-striatal circuitry relevant to compulsivity – the SMA and ACC – will be more evident in OCD and probably expressed in the healthy population as a function of the transdiagnostic dimension of compulsivity. Specifically, we predicted relationships in the balance between Glu and GABA function in these frontal areas and compulsive and habitual tendencies, as well as clinical symptoms which are an extreme expression of compulsive behaviour. MRS and behavioural data were thus collected from 30 healthy subjects and 31 participants with OCD (clinical and demographic measures for both groups are shown in Table 3).

## Results

### Differential balance between excitatory and inhibitory neurotransmitters in healthy and OCD brain

**Fig. 1:**
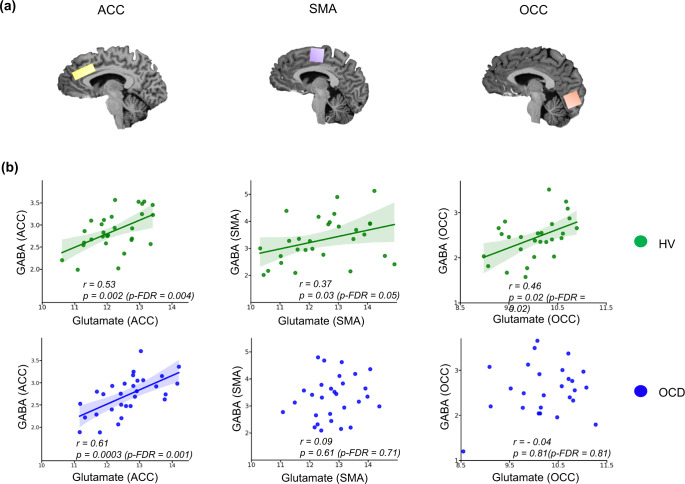
Relationship between Glutamate and GABA in the anterior cingulate cortex, the supplementary motor area and the occipital cortex of the healthy and OCD brain. The glutamate and GABA levels, expressed in parts per million (ppm), were measured in voxels placed in (**a**) anterior cingulate cortex (12 × 20 × 33 mm^3^), in yellow, supplementary motor area (20 × 20 × 20 mm^3^) in purple, and occipital cortex (20 × 20 × 20 mm^3^) in orange, of (**b**) healthy participants in green, and participants with OCD in blue. The line of best fit is shown with the 95% confidence intervals for the regression estimate in translucent bands around the regression lines. All metabolites were normalised using (Cr + PCr), corrected for grey and white matter and cerebral spinal fluid of each individual voxel, within subjects. For the ACC voxel the sample size for Glu and GABA in the OCD group was (*n* = 30) and in HV (*n* = 30). For the SMA voxel the sample size for Glu in the OCD group was (*n* = 31) and in the HV it was (*n* = 30), for GABA in the OCD group it was (*n* = 30) and in the HV the sample size was (*n* = 29). Lastly, for the OCC voxel the sample size for Glu in the OCD group was (*n* = 30) and in the HV it was (*n* = 28), for GABA in the OCD group it was (*n* = 27) and in the HV the sample size was (*n* = 29). All relationships were studies using a two-tailed Pearson test. The data for this figure are provided in the Source Data file. Acronyms: ACC anterior cingulate cortex, SMA supplementary motor area, OCC occipital cortex, GABA γ-amino-butyric acid, *p*-FDR *p*-value corrected for False Discovery Rates^98^, *r* Pearson’s *r* correlation coefficient, HV Healthy Volunteers, OCD Obsessive Compulsive Disorder.

Figure 1 shows the positive relationships between Glu and GABA in the ACC for the OCD and healthy subjects separately (OCD + healthy subjects: Pearson’s *r* = 0.51, *p* = 0.00003, *p*-FDR = 0.0002), whereas only healthy volunteers showed a positive relationship in SMA (Pearson’s *r* = 0.37, *p* = 0.04, *p*-FDR = 0.05) and OCC (Pearson’s *r* = 0.46, *p* = 0.01, *p*-FDR = 0.01). However, participants with OCD showed this relationship in neither SMA (Pearson’s *r* = 0.09, *p* = 0.61, *p*-FDR = 0.71), nor occipital cortex (Pearson’s *r* = −0.04, *p* = 0.81, *p*-FDR = 0.81). Thus, the Glu/GABA relationship is a likely cortex-wide finding in the healthy group as opposed to the OCD group who showed an imbalance in the relationship between these metabolites in both SMA and OCC. Figure 1b depicts these results separately for healthy volunteers and participants with OCD.

In addition to the correlation analysis above, in order to substantiate the conclusions further, a confirmatory 6 stage hierarchical linear regression model was built to predict Glu levels from GABA concentrations, while controlling for Group (OCD vs HV) and Voxel (OCC, ACC and SMA regions). Analysis of variance was used to select the best model (model4: *Glu*~GABA*Group+Voxel), predicting around 65% of variability in Glu levels (*F* (5,167) = 65.10, *p* < 2.2e−16. Table 1 shows the output summary for this model. There was a significant effect of voxel (ACC, SMA, OCC), a significant effect of group (OCD vs HV), and a trend for the group and voxel interaction. These findings provide clear support for the claim that the relationship between Glu and GABA is altered in participants with OCD, though only modest support for the claim that this effect varies by voxel. All model outputs are described in detail in Supplementary Information, including the R code.

**Table 1:** Summary of the best model of the Hierarchical Linear Regression analysis using GABA to predict Glu levels while controlling for Group and Voxels: Glu~GABA*Group *+* *Voxel*

Coefficients	β-estimates	Std.Error	*t*-test	*p*-value
Intercept	10.48	0.46	22.63	<2e−16
GABA	1.82	0.45	4.00	9.23e−05
Group (OCD)	1.33	0.58	2.30	0.02
Voxel (OCC)	−2.09	0.16	−13.06	<2e−16
Voxel (SMA)	0.01	0.16	0.07	0.98
GABA:Group(OCD)	−1.05	0.58	−1.81	0.07

The data for this table are provided in the Source Data file and the code is provided in the Supplementary Information. For the ACC voxel, the sample size for Glu and GABA in the OCD group was (*n* = 30) and in HV (*n* = 30). For the SMA voxel, the sample size for Glu in the OCD group was (*n* = 31) and in the HV it was (*n* = 30), for GABA in the OCD group it was (*n* = 30) and in the HV the sample size was (*n* = 29). Lastly, for the OCC voxel, the sample size for Glu in the OCD group was (*n* = 30) and in the HV it was (*n* = 28), for GABA in the OCD group it was (*n* = 27) and in the HV the sample size was (*n* = 29).

*GABA* γ-amino-butyric acid, *Glu* glutamate, *SMA* supplementary motor area, *OCC* occipital cortex, *OCD* obsessive-compulsive disorder.

### Comparing neurometabolite levels between HV and OCD groups

Within each voxel, the levels of GABA, Glu, Glu/GABA, Gln, Glx (glutamate + glutamine) and NAA (N-acetylaspartate, a measure of neuronal integrity)^28^ were compared between groups (Table 2). Participants with OCD showed significantly higher levels of Glu (independent sample *t*-test(58) = 2.08, *p* = 0.02, *p*-FDR = 0.04, Cohen’s *d* = *0.53*, 95% CI[0.016, 0.83]), Glu:GABA ratio (Mann–Whitney *U* = 618, *p* = 0.006, *p*-FDR = 0.02, η_p_^2^ = 0.10, 95% CI[0.05, 0.70]), and Glx (independent sample *t*-test(58) = 2.13, *p* = 0.02, *p*-FDR = 0.05, Cohen’s *d* = *0.55*, 95% CI[0.03, 1.13]) within the ACC voxel. When controlling for NAA levels as a control for neuronal integrity, GABA levels in ACC become significantly lower in OCD compared with the healthy subjects (ANCOVA *F*-test (1, 57) = 4.55, *p* = 0.03, *p*-FDR = 0.04, η_p_^2^ = 0.074, 95% CI[0.012, 0.35]), whereas, OCC Glu level differences become non-significant (*p* = 0.20). There were no differences between metabolite levels within the SMA between OCD and healthy volunteers. The MRS quality metrics per metabolite and tissue compositions per voxel are presented in Table S1(Supplementary Information).

**Table 2:** This table depicts the mean ± standard deviations of all neurometabolites in OCD and HV, displayed for anterior cingulate cortex, supplementary motor area, and occipital cortex and compared between groups

Metabolites	OCD group (M ± SD)	HV group (M ± SD)	*t*-test	DF	*p*-value	*d*	*p-FDR*	NAA-corr F-test	DF	*p*-value	η_p_^2^	*p-FDR*
ACC												
GABA	2.71 ± 0.43	2.86 ± 0.42	−1.29	58	0.10	−0.33	0.15	4.58	(1,57)	**0.03**	0.074	**0.04**
Gln	3.37 ± 0.58	3.21 ± 0.48	1.18	58	0.12	0.30	0.14	1.11	(1,57)	0.29	0.019	0.29
Glu	12.63 ± 0.82	12.20 ± 0.74	−2.08	58	**0.02**	0.53	**0.04**	5.66	(1,57)	**0.02**	0.090	**0.03**
Glx (Glu + Gln)	16.00 ± 1.13	15.41 ± 0.98	2.13	58	**0.02**	0.55	0.06	5.68	(1,57)	**0.02**	0.091	0.05
NAA	12.63 ± 0.94	12.51 ± 0.92	0.48	58	0. 61	0.13	0. 61	NA	NA	NA	NA	NA
Glu/GABA	4.73 ± 0.64	4.34 ± 0.61	618^U^	NA	**0.006**	0.10${\,\!}^{{\upeta_{\rm p}}{\,\!}^2}$	**0.03**	**8.65**	(1,57)	**0.005**	0.13	**0.02**
SMA												
GABA	3.41 ± 0.97	3.31 ± 0.83	450^U^	NA	0.41	0.0008${\,\!}^{{\upeta_{\rm p}}{\,\!}^2}$	1.00	0.04	(1,56)	0.84	0.001	1.00
Gln	2.53 ± 0.93	2.40 ± 0.85	0.56	57	0.29	0.14	0.69	0.28	(1,56)	0.59	0.005	1.00
Glu	12.64 ± 0.97	12.50 ± 1.28	0.50	59	0.45	0.13	0.61	0.03	(1,58)	0.86	0.000	0.86
Glx (Glu + Gln)	15.34 ± 1.38	14.90 ± 1.82	1.03	57	0.30	0.27	1.00	0.80	(1,56)	0.37	0.014	1.00
NAA	15.37 ± 1.42	15.14 ± 1.43	0.64	59	0.26	0.16	0.78	NA	NA	NA	NA	NA
Glu/GABA	4.00 ± 1.09	3.97 ± 0.98	445^U^	NA	0.44	−0.0004${\,\!}^{{\upeta_{\rm p}}{\,\!}^2}$	0.88	0.07	(1,56)	0.79	0.001	1.00
OCC												
GABA	2.55 ± 0.55	2.48 ± 0.48	0.51	54	0.30	0.13	0.36	0.25	(1,53)	0.62	0.005	0.77
Gln	3.88 ± 0.64	4.05 ± 0.52	−1.12	56	0.13	−0.29	0.25	1.15	(1,55)	0.29	0.021	0.72
Glu	10.39 ± 0.78	10.08 ± 0.60	1.70	54	0.09	0.44	0.25	1.65	(1,55)	0.20	0.029	1.0
Glx (Glu + Gln)	14.27 ± 1.01	14.01 ± 1.14	0.93	56	0.17	0.24	0.25	0.43	(1,57)	0.51	0.007	0.85
NAA	14.79 ± 1.05	14.57 ± 1.06	0.84	58	0.40	0.21	0.40	NA	NA	NA	NA	NA
Glu/GABA	4.23 ± 1.02	4.18 ± 0.80	1.46	54	0.15	0.05	0.25	0.03	(1,53)	0.85	0.001	0.85

To minimise concentration levels being contaminated with noise, and to avoid excluding abnormal data points that are population specific and may be clinically relevant, % Cramér-Rao Lower Bound and concentration levels outside the mean ± 2 SD were excluded, following procedure from Frangou et al.^97^ and as suggested by Kreis^80^. All metabolites were normalised using (Cr + PCr), corrected for grey and white matter and cerebral spinal fluid of each individual voxel, within subjects. The *F*-test values are the results of an ANCOVA using NAA as covariate to correct as a measure of neuronal integrity. The bold formatting shows the significant *p*-values and the metabolites that were measured for each significant test. For the ACC voxel the sample size for Glu and GABA in the OCD group was (*n* = 30) and in HV (*n* = 30). For the SMA voxel the sample size for Glu in the OCD group was (*n* = 31) and in the HV it was (*n* = 30), for GABA in the OCD group it was (*n* = 30) and in the HV the sample size was (*n* = 29). Lastly, for the OCC voxel the sample size for Glu in the OCD group was (*n* = 30) and in the HV it was (*n* = 28), for GABA in the OCD group it was (*n* = 27) and in the HV the sample size was (*n* = 29). All tests were two sided except for ACC and SMA glutamate, glutamine and GABA level comparisons across groups. The data for this table are provided in the Source Data file.

*ACC* anterior cingulate cortex, *SMA* supplementary motor area, *OCC* occipital cortex, *GABA* γ-amino-butyric acid, *Glu* glutamate, *Gln* glutamine, *Glx* Glu + Gln, *NAA* N-acetylaspartate, *Cr* creatine, *PCr* Phosphocreatine, *t* independent sample *t*-test, *U* Mann–Whitney U test, *F* Analysis of Covariance, *NAA-c* corrected for NAA levels, *M* mean, *SD* standard deviation, *HV* healthy volunteers, *OCD* obsessive-compulsive disorder, *p-FDR* *p*-value corrected for False Discovery Rates^98^, *DF* degrees of freedom, *d* Cohen’s *d*, *η*_*p*_^*2*^ partial eta-square.

### Relationship between compulsivity and SMA brain metabolites

**Fig. 2:**
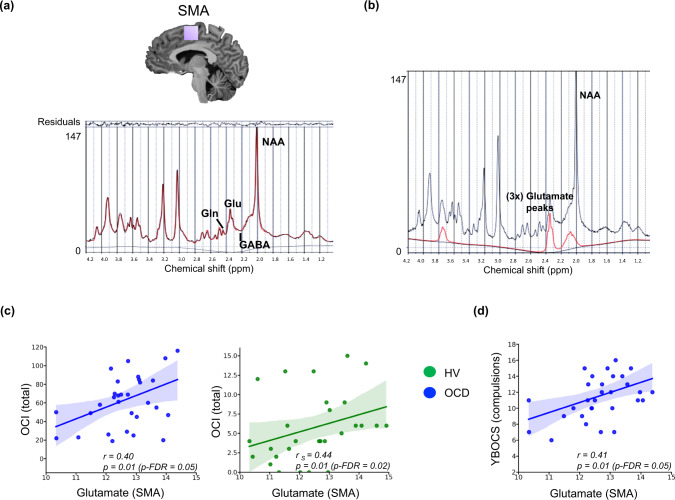
Relationships between compulsivity and Glu levels in SMA. **a** Shows the LCModel analysis of in vivo ^1^H MR spectra acquired from a healthy participant at 7T (semi-LASER, echo time/repetition time = 26/5000 ms, from a 20 × 20 × 20 mm voxel placed bilaterally at supplementary and pre-supplementary motor areas, located at medial portion of Brodmann area 6), **b** presents the fitted model for Glu, in red, while the acquired spectrum is plotted in black. **c** Demonstrates the relationships between Glu levels in SMA and obsessive-compulsive symptoms as measured with the self-administered OCI scale in the individuals with OCD and in healthy subjects (OCI was missing for 2 HV’s), while **d** depicts the relationship between the clinician rated YBOCS scores and Glu levels in SMA in the OCD group. The blue colour represents OCD patients, whereas green depicts the data for healthy subjects. The line of best fit is shown with the 95% confidence intervals for the regression estimate in translucent bands around the regression lines (for all figures in **c** and **d**). For figure **c** the sample size for the OCD subjects was (*n* = 30) and for the HV group it was (*n* = 29), for figure **d** the OCD sample size was (*n* = 31). All relationships were studies using a two-tailed Pearson test. The data for this figure are provided in the Source Data file. Acronyms: SMA supplementary motor area, GABA γ-amino-butyric acid, Glu glutamate, Gln glutamine, NAA N-acetylaspartate, ppm parts per million, *r* Pearson’s *r* correlation coefficient, *r*_s_ Spearman’s rank correlation coefficient, *p*-FDR *p*-value corrected for False Discovery Rates^98^, OCI Obsessive Compulsive Inventory, YBOCS Yale Brown Obsessive Compulsive Scale, HV healthy volunteers, OCD obsessive-compulsive disorder.

Figure 2a shows the SMA voxel and its MRS spectrum for all metabolites and Fig. 2b displays the fitted model for Glu, for one representative individual. There was a positive relationship between compulsive tendencies using the self-administered Obsessive-Compulsive Inventory (OCI)^29^ and Glu levels in SMA for the entire sample (i.e., OCD + healthy subjects: Spearman’s *r* = 0.28, *p* = 0.02, *p*-FDR = 0.03). Participants with OCD exhibited, as expected, significantly higher OCI scores (*p* < 0.001), and since the OCI scores had different distributions within each group, the relationships between the Glu concentrations and OCI were separately analysed per group. Both were significant (see Fig. 2c): OCD (with normally distributed OCI): Pearson’s *r* = 0.40, *p* = 0.01, *p*-FDR = 0.05, and healthy volunteers (with non-parametrically distributed OCI): Spearman’s *r* = 0.44, *p* = 0.01, *p*-FDR = 0.02). In line with these findings, the compulsions subscale of the clinician rated Yale Brown Obsessive Compulsive Scale (YBOCS)^30^ was also correlated with SMA Glu levels in the OCD group (Pearson’s *r* = 0.41, *p* = 0.01, *p*-FDR = 0.05; Fig. 2d). Supplementary Fig. S4 (Supplementary Information) depicts individual spectra for Gln, GABA, and NAA within a representative SMA voxel. With regard to ACC neurometabolites, no correlation was found between ACC Glu:GABA ratio and obsessive compulsive symptoms as measured with OCI and YBOCS in the OCD group (Pearson’s *r* = −0.16, *p* = 0.39; *r* = −0.18, *p* = 0.33, respectively for YBOCS compulsions and OCI total score).

### Neural correlates of habitual responding: SMA and ACC Glu:GABA ratios implicated in habitual control

**Fig. 3:**
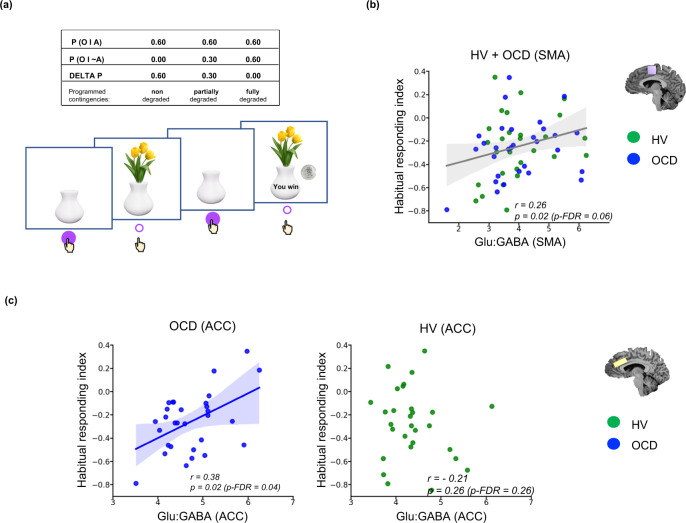
Relationship between habitual responding as measured with the contingency degradation task and Glu:GABA ratios in ACC and SMA. **a** Depicts the stimuli from the contingency degradation task^31^ and its task design. Participants are shown a white empty vase on the screen, which fills with flowers when participants press the space bar, and which has a 60% chance of being associated with winning 20 pence. On degraded trials, this 60% positive contingency is reduced to 0% (full degradation) or 30% (partial degradation), by non-response contingent presentations of reward. **b** Shows the positive relationship between an index of habitual responding, the difference between full and non-degraded conditions (higher means a bias towards habitual control) and Glu:GABA ratio in SMA of the entire sample of OCD and healthy volunteers (the grey fitted line). **c** Shows the positive relationship between the index of habitual responding and the anterior cingulate cortex for each group separately. The blue colour represents OCD patients, whereas green depicts the data for healthy subjects. The line of best fit is shown with the 95% confidence intervals for the regression estimate in translucent bands around the regression lines. For figure **b** the total sample size was (*n* = 59): 30 OCD and 29 healthy participants. For figure **c** the sample size for the OCD group was (*n* = 30) and in HV (*n* = 30). All relationships were studied using a two-tailed Pearson test. The data for this figure are provided in the Source Data file. Acronyms: SMA supplementary motor area, ACC anterior cingulate cortex, GABA γ-amino-butyric acid, Glu glutamate, *r* Pearson’s r correlation coefficient, *p*-FDR *p*-value corrected for False Discovery Rates^98^, *O* outcome, *P* probability, HV healthy volunteers, OCD obsessive-compulsive disorder.

A contingency degradation task^31^ was used to measure the tendency towards habitual (stimulus-response) as opposed to goal-directed (action-outcome) responding^23^. The task comprises 3 conditions with varying probabilities (0.60, 0.3, 0.0: see Methods) of gaining rewards of 20 pence (£0.2) by responses of pressing a space bar. These contingencies between instrumental responses and reward outcomes were obtained by varying the provision of free rewards where no response was required to earn the monetary rewards. We also required subjects to make regular causal judgements concerning their perception of the relationship between their responses and the reward outcomes, in order to gauge their understanding of the contingencies (see Fig. 3a). As expected (see also Ersche et al. 2021^31^), responding declined significantly (though equivalently) in both groups in the degraded conditions (Supplementary Fig. S2, Supplementary Information), consistent with the programmed reinforcement contingencies. The differences between the two non-degraded and degraded conditions are assumed to reflect the balance between goal-directed and habitual control, the smaller the decrement being more consistent with the latter. Subjective judgements of contingencies were highly and positively correlated with the behavioural measures for the entire sample (Supplementary Fig. S3b, Supplementary Information). A habitual responding index was created by subtracting responses made in the non-degraded condition (i.e., probability of 0.60) condition from those with a low probability (0.3 or 0.0) of actions gaining rewards. This means that increasingly negative values indicate a greater tendency towards goal-directed behaviour, whereas less negative and positive values represent bias towards habitual control.

The entire sample showed a significant positive relationship between the habitual responding index and SMA Glu:GABA ratio (Pearson’s *r* = 0.26, *p* = 0.02, *p*-FDR = 0.06; Fig. 3b). This means that higher values of Glu and/or lower values of GABA were associated with a greater habitual tendency. Moreover, participants with OCD showed this same relationship within the ACC voxel as well (Pearson’s *r* = 0.38, *p* = 0.02, *p*-FDR = 0.04; Fig. 3c). Note that the habitual responding index was equivalent in both groups (Supplementary Fig. S3a, Supplementary Information).

## Discussion

This 7T ^1^H-MRS study has demonstrated that compulsivity and clinical compulsive symptoms are related to neurochemical indices in the anterior cingulate cortex and supplementary motor area of the frontal lobes, suggestive of an altered excitatory/inhibitory (E/I) balance between the neurometabolites Glu and GABA in these regions. Moreover, this neurochemical imbalance, as hypothesised, was also related to a measure of habitual responding versus goal-directed control over behaviour in these regions, relevant to theories proposing that this bias underlies compulsive behaviour in clinical disorders of obsessive-compulsive disorder and drug addiction^3,32^.

We studied neurochemical correlates of compulsivity in healthy volunteers and in a group of adult participants with OCD, using 7T ^1^H-MRS to measure the proton spectrum with high signal-to-noise ratios in three brain regions of prior interest (SMA, ACC and OCC). We used clinician-rated (Y-BOCS) and self-rated (OCI) symptom questionnaires as well as behavioural performance data on a test of habitual vs goal-directed responding, to define compulsivity and its underlying cognitive traits. We chose to focus on MRS profiling of the ACC and SMA because both have been previously implicated in the neural circuitry of compulsivity (see Introduction).

Glu and GABA levels were significantly correlated in all regions studied in healthy volunteers, but not in the OCD group, suggesting that a dysfunctional balance may contribute to pathology. In fact, participants with OCD had significantly higher levels of Glu and lower levels of GABA in the ACC and a higher Glu:GABA ratio in that region. These significant changes were found in *both* of these neurometabolites in the same study in adult OCD, possibly reflecting the greater sensitivity of 7T ^1^H-MRS. Previous studies at lower magnet strengths have found evidence for greater Glu concentrations (though less specifically, Glu + Gln measured together as Glx) in this region of the ACC^33,34^. Naaijen et al. also found increased Glu in the anterior cingulate in children with OCD or autism spectrum disorder using a 3T scanner^35^, suggesting that these changes may occur early in life. On the other hand, several studies have found no significant effects in prefrontal cortex (PFC) regions^12,13,36–39^ or even reductions^40,41^. For GABA, Simpson et al.^12^ also found reductions in a medial PFC voxel including the ACC, and Zhang et al.^38^ reported a decreasing trend in the ACC and a reduction in the orbitofrontal cortex. However, a more recent study found increased GABA levels in the ACC^13^.

We found no significant changes in Glu or GABA in the occipital cortex in OCD, or any evidence of correlation with behaviour or symptoms despite suggestive evidence from some other neuroimaging studies of changes in this region^42–47^. Our findings for OCD were therefore specific to the anterior neocortex.

The bidirectional changes in Glu and GABA neurometabolites demonstrated here in the ACC of participants with OCD are an important addition to MRS findings in OCD because it sheds light on this inconsistent literature^10,11^. It is possible that this inconsistency has stemmed from the use, in many cases, of small sample sizes, different populations of participants with OCD (with co-morbidities such as skin-picking and depression^48^) as well as evidence of impaired neuronal integrity (in studies finding concomitant changes in NAA^38,49–52^) and variation in voxel placements. Moreover, the literature overall has hitherto also perhaps lacked the resolution to quantify metabolites with smaller peaks such as GABA, Glu and Gln with a higher accuracy and a superior signal-to-noise ratio^11^. Another strength of this study is that we have adhered to the minimum guidelines for the reporting of MRS methods and results, and followed the Lin et al. (2020) checklist in presenting the standardised description of MRS hardware, data acquisition, analysis and quality assessment^53^.

The changes we found in ACC Glu and GABA were shown to be independent of the grey and white matter and CSF composition within each individual voxel for each subject. We additionally controlled for neuronal integrity via NAA changes, this resulting in more significant depletion of ACC GABA levels in the OCD group.

Despite the lack of absolute changes in Glu in the SMA in OCD, we found that clinical symptoms (YBOCS-compulsivity subscale) were significantly positively related to the SMA Glu levels, which adds weight to recent suggestions that increased SMA activity is a neuroendophenotype for OCD^6^. In addition, compulsivity trait (OCI) was also related to changes in SMA Glu levels, both in healthy volunteers and participants with OCD, consistent with the notion of a transdiagnostic dimension characterising compulsive behaviour related to connectivity of frontal regions^2^. Finally, the measure of habitual responding was reflected by the Glu:GABA ratio in the SMA in both healthy volunteers and participants with OCD, and additionally in the ACC in the OCD group. These findings extend our existing understanding of how fronto-striatal loops control the balance between goal-directed behaviour and habits, and how this balance may be related to the generation of compulsive responding.

Previous MRS studies have used the ratio of excitatory (Glu) and inhibitory (GABA) neurotransmitters as a proxy index of E/I balance in cortical networks and recent work found a positive correlation between GABA and Glx in medial parietal cortex^54^, consistent with electrophysiological evidence^55^. However, such relationships are controversial and another study, performed at 3T failed to show positive correlations between Glx and GABA in visual and motor cortex^56^. Nevertheless, in this 7T study we were able to demonstrate in healthy volunteers using an hierarchical linear regression model in addition to correlational analysis, robust positive relationships between Glu and GABA in ACC, supplementary motor area and occipital cortex, suggesting that these indices may be more stable indices of E/I balance than Glx/GABA. The absence of a positive Glu/GABA relationship in OCD within the SMA is consistent with other evidence in this study that clinical symptoms are associated with Glu levels in the SMA and with substantial evidence in OCD of BOLD hyperactivity, increased functional connectivity, exaggerated error-related and readiness potentials in the SMA as well as in the ACC^6,19,21,57^.

In contrast, SMA Glu level was related significantly to the compulsion sub-score for the YBOCS in participants with OCD, even though these levels were not different from healthy volunteers. As discussed above, the relevant difference between OCD and healthy volunteers may actually be in the lack of E/I balance. The findings are consistent with other data indicating a significant relationship between right SMA BOLD activity and YBOCS score in OCD^6^. Of course, this does not necessarily indicate that the SMA is the origin of OCD symptoms, especially as other studies have shown similar correlations with fronto-striatal structure and connectivity^58,59^, but it does place the SMA importantly within that network.

A major developmental study of compulsivity trait using magnetisation transfer (MT) imaging to quantify network connectivity in adolescents found that a composite compulsivity score (OCI plus Padua scale) was related to reduced growth of the MT trajectories in fronto-striatal circuity, most markedly in dorsolateral and dorsomedial prefrontal regions, including the SMA, consistent with our findings in this region, and further supporting a possible causal role of the changes in neural network function in OCD^2^. Although we also found a significant relationship between OCI and SMA Glu (*r* = 0.28, *p* < 0.02) for the entire sample (i.e., OCD and healthy volunteers), separate analyses of two sub-groups in fact show even more significant relationships (Fig. 2c). This is probably due to OCI scores being normally distributed in OCD group but not in healthy volunteers, given our recruitment criterion of <42 OCI scores in the latter. In general, the finding is consistent with the hypothesis that compulsive behaviour can exist along a continuum and be mediated by similar neural networks.

What has perhaps been lacking in previous studies of the neurocognitive substrates of compulsivity has been a theoretical basis for the psychological mechanisms underlying this form of behaviour. Evidence has been reviewed supporting a previous hypothesis suggesting that compulsion in OCD can result from an imbalance in competing neural systems governing instrumental goal-directed and habitual control of behaviour with a bias to the latter^60^. Part of this evidence has depended on a test of contingency degradation whereby instrumental actions are uncoupled from their outcomes by weakening their predictive correlation. Thus, participants with OCD continued to respond in some conditions despite showing normal subjective causal judgement of the contingencies, presumably as a consequence of greater habitual control over performance^61^. Consequently, in this study we also included a simplified contingency degradation test modified for use in neuroimaging studies and previously shown to be sensitive to deficits in stimulant drug abusers, as well as being validated by a subjective habit questionnaire^31^. Our habitual responding index from the contingency degradation test was significantly related to Glu:GABA ratio in the SMA for the entire sample. This is consistent with evidence from both human neuroimaging and animal studies^23,25,62^ of a possible role for this premotor cortical region, in conjunction with the putamen to which it projects, as part of the so-called ‘habit system’. However, a specific role for the SMA, as distinct from other premotor regions in habitual control, has hitherto not been much researched (e.g.,^63,64^); although a recent article^24^ specifically identified such a role for this region. Previous functional neuroimaging studies of action-outcome contingency learning have implicated a ventromedial prefrontal cortex-caudate circuitry, whereas action-independent outcomes were associated with inferior frontal gyrus^65,66^. The inferior frontal gyrus, in conjunction with the pre-SMA, has also been implicated in behavioural inhibition^67,68^, including in OCD^69^. This suggests that our findings may relate to the expression of habitual responding, as the SMA (and pre-SMA) are widely believed to mediate the initiation and inhibition of voluntary behaviour^70,71^. Further supporting a cortico-striatal ‘habit’ system, Ersche et al. found that individuals with cocaine use disorder with elevated habitual scores exhibited reduced Glu in the putamen using the same contingency degradation test paradigm and the same 7T scanner with similar sequence parameters^31^.

There was also a relationship between the index of habitual responding and ACC Glu: GABA ratio in participants with OCD alone. This is consistent with recent evidence of effects of pharmacologically stimulating activity of BA24 (dorsal ACC) in the marmoset monkey with a Glu reuptake inhibitor, although deficits were also found when a mixture of GABA agonists (muscimol and baclofen) were infused into this region (the only one of several other frontal areas examined to show these effects)^62^. However, we found no relationship for the ACC and habitual responding in HVs. Therefore, there were differences as well as the similarities (in the SMA) in the neural substrates of habitual control between participants with OCD and healthy volunteers. These results are more generally consistent with other findings suggesting that the SMA and anterior cingulate have strong reciprocal connections that act to sustain each other’s activity, and that this interaction is mediated during movement preparation according to the readiness potential amplitude^57^, which is enhanced in OCD^72^.

Overall, the findings are relevant to the hypothesis that participants with OCD exhibit a bias away from goal-directed behaviour towards habitual control. However, in this particular test of contingency degradation there were actually no significant differences in performance, which contrasts with OCD impairments previously shown in the more elaborate test paradigm used earlier^61^. This means that no behavioural support was found for the hypothesis that participants with OCD exhibit weaker goal-directed behaviour or strengthened habitual responding in this test procedure, simplified for the purpose of neuroimaging. Nevertheless, the neural mediation of habitual control in OCD additionally implicated the ACC, in contrast to HVs (Fig. 3c). The ACC may have been recruited in OCD because of its known role in the mediation of action-outcome learning and error prediction, especially under uncertainty^16,17,19,73^. This recruitment possibly reflects a functional compensation in the participants with OCD, especially given the classical role of the ACC in response to error-monitoring performance^73–76^. This possibility of functional compensation in OCD is consistent with previous evidence from both MRS^37^ and error-related negativity studies^77,78^.

Limitations of our study include the medication status of the participants with OCD, although we assume that this cannot account for the relationships shown with clinical symptoms or in the healthy volunteers. Moreover, SSRIs have generally been shown to reduce Glu^79^ and so are unlikely to have produced the enhanced Glu levels seen here. Additionally, several of the previous studies showing changes in neurometabolites employed unmedicated OCD samples^7,12,52^. Sample size is a consideration although we believe our study was adequately powered, especially given the improved sensitivity of 7T ^1^H-MRS. We rigorously controlled for multiple correlations using FDR. As recommended for clinical studies of MRS we included extreme values as long as they were within 2SD from the group average^80^.

Finally, we accept that our analysis is entirely correlational and more rigorous testing of causality would probably have to depend on assessing effects of suitable interventions. This could potentially be therapeutically advantageous in considering treatments based on pharmacological manipulation of neurotransmitter functions. This might include regulation of Glu^7–9^ or GABA activity; a recent study of therapeutic effects of ketamine in OCD showed changes in ACC GABA^81^. Alternatively, neuromodulatory interventions such as deep brain stimulation or non-invasive transcranial magnetic stimulation^26,27^ could be employed with the same aim of restoring normal E/I balance to specific fronto-striatal or cingulate-striatal circuits.

## Methods

### Participants

This study included 30 healthy volunteers and 31 participants with OCD who were fluent English speakers and were matched for age, gender and IQ. Table 3 shows the demographic and clinical characteristics of both groups.

**Table 3:** Clinical and demographic information for healthy volunteers and participants with OCD

	OCD (M ± SD)	HV (M ± SD)	*t*-test	DF	*p*	*d*	*95% CI*
*N*	31	30					
Age (years)	30.79 ± 9.99	32.16 ± 12.98	−0.46	59	0.64	−0.12	[−7.29, 4.55]
Gender (% female)	55%	50%	0.143 ${\,\!}^{{{{\mathrm{X}}}}^{2}}$	1	0.70	−0.05 ^Phi^	[0.30, 2.25]
Education (years)	15.35 ± 2.85	17.23 ± 2.72	−2.62	59	0.01	−0.67	[−3.31, −0.44]
Verbal IQ	110.21 ± 6.13	113.11 ± 7.75	−1.62	59	0.11	−0.41	[−6.48, 0.67]
MADRS	17.65 ± 11.01	5.07 ± 3.78	6.00	37.20	<0.001	1.52	[8.33, 16.82]
STAI-S	37.65 ± 9.12	27.70 ± 6.83	4.80	59	<0.001	1.23	[5.80, 14.08]
STAI-T	56.55 ± 11.03	37.13 ± 10.80	6.94	59	<0.001	1.77	[13.81, 25.01]
YBOCS total	22.94 ± 5.90	NA	NA	NA	NA	NA	NA
YBOCS obsessions	11.45 ± 3.21	NA	NA	NA	NA	NA	NA
YBOCS compulsions	11.52 ± 2.97	NA	NA	NA	NA	NA	NA
OCI total	63.32 ± 30.37	6.07 ± 5.80	924^U^	NA	<0.001	−0.73${\,\!}^{{\upeta_{\rm p}}{\,\!}^2}$	[45.0, 66.0]
OCI washing	10.48 ± 9.86	1.00 ± 1.39	793^U^	NA	<0.001	−0.38${\,\!}^{{\upeta_{\rm p}}{\,\!}^2}$	[2.0, 11.0]
OCI checking	13.06 ± 8.28	1.23 ± 1.45	868^U^	NA	<0.001	−0.58${\,\!}^{{\upeta_{\rm p}}{\,\!}^2}$	[9.0, 14.0]
OCI doubting	6.26 ± 3.56	0.23 ± 0.62	874^U^	NA	<0.001	−0.63${\,\!}^{{\upeta_{\rm p}}{\,\!}^2}$	[4.0, 9.0]
OCI ordering	7.29 ± 5.24	1.30 ± 1.49	836^U^	NA	<0.001	−0.48${\,\!}^{{\upeta_{\rm p}}{\,\!}^2}$	[3.0, 7.0]
OCI obsessions	14.42 ± 8.11	0.73 ± 1.20	896^U^	NA	<0.001	−0.66${\,\!}^{{\upeta_{\rm p}}{\,\!}^2}$	[11.0, 16.0]
OCI hoarding	3.16 ± 2.86	0.93 ± 1.72	696^U^	NA	<0.001	−0.19${\,\!}^{{\upeta_{\rm p}}{\,\!}^2}$	[1.0, 3.0]
OCI neutralisation	8.65 ± 5.77	0.73 ± 0.94	878^U^	NA	<0.001	−0.60${\,\!}^{{\upeta_{\rm p}}{\,\!}^2}$	[4.0, 10.0]
IUS	84.61 ± 22.85	54.37 ± 16.93	5.88	59	<0.001	1.50	[19.91, 40.58]
HTQ compulsivity	23.87 ± 4.21	13.60 ± 5.56	8.15	59	<0.001	2.08	[7.75, 12.81]

The sample size for OCI in healthy group is 28 because two participants did not provide these data, for the rest of the data there were 30 healthy subjects and 31 participants with OCD. The tests used for age, gender, education and verbal IQ were two-sided, and the rest of the variables were tested using a one-sided test. The data for this table are provided in the Source Data file.

*NA* Not Applicable, *CI* Confidence Interval, *MADRS* Montgomery- Asberg Depression Rating Scale, *STAI-T* State Trait Anxiety Inventory- Trait, *STAI-S* State Trait Anxiety Inventory- State, *YBOCS* Yale Brown Obsessive Compulsive Scale, *OCI* Obsessive-Compulsive Inventory, *IUS* Intolerance of Uncertainty Scale, *HTQ* Habitual Tendencies Questionnaire, *HV* healthy volunteers, *OCD* obsessive compulsive disorder, *DF* degree of freedom, *U* Mann–Whitney *U* test for not normally distributed data, *X*^2^ Chi-Square test for categorical data, *η*_*p*_^*2*^ partial eta-square (a measure of effect size for the U test), *Phi* a measure of effect size for the *X*^2^ test, *d* Cohen’s *d*, *CI* Confidence Interval of the *t*-test.

Healthy individuals were recruited from the community, were all in good health, unmedicated and had no history of neurological or psychiatric conditions. Participants with OCD were recruited through an approved advertisement on the OCD action website (www.ocdaction.org.uk) and local support groups and via clinicians in East Anglia. All participants received monetary compensation for taking part in this study. Participants with OCD were screened by a qualified psychiatrist of our team (A.S.) to confirm a primary OCD diagnosis. Additionally, the Mini International Neuropsychiatric Inventory (MINI) was used to confirm the absence of any psychiatric conditions in both groups. Within the OCD group, OCD symptom severity and characteristics were measured using Yale Brown Obsessive Compulsive Scale (YBOCS)^30^. In both OCD and healthy participants, mood status was assessed using the Montgomery-Asberg Depression Rating Scale (MADRS)^82^, anxiety levels were evaluated using the State-Trait Anxiety Inventory (STAI)^83^, and verbal IQ was quantified using the National Adult Reading Test (NART)^84^. All individuals included in the OCD group had a diagnosis of OCD (as per the DSM-V criteria), and exhibited anxiety and depressive symptoms. OCD participants with comorbid major depressive disorder or Generalised Anxiety Disorder, or any additional axis-I disorders, were not included in the study. We included only OCD participants who presented a total YBOCS score higher than 12^85^. We included 4 participants with OCD within the lower end of the YBOCS, i.e., mild symptomatology (scores between 12 and 15) to enable a broader range of symptom severity, important for the correlational analyses. Six OCD subjects were unmedicated, and out of the 25 medicated patients, 2 were additionally on beta blockers, 1 on clomipramine, 1 on olanzapine and the rest were on SSRIs monotherapy. General exclusion criteria during recruitment for both groups were substance dependence, neurological or medical illnesses or head injury. All participants had normal or corrected- to normal vision and hearing. As the groups were matched for gender and in almost all participants (one OCD participant) gender and sex were the same, we did not control for them in our analysis.

All participants completed the following additional self-report questionnaires:

- Obsessive-Compulsive Inventory^29^: a standardised self-report measure of obsessive-compulsive symptoms
- Intolerance of Uncertainty Scale^86^: a standardised self-report measure on the unpleasantness of uncertainty
- The compulsivity subscale of the Habitual Tendencies Questionnaire (HTQ)^87^

This study was approved by the East of England - Cambridge South Research Ethics Committee (REC 16/EE/0465). All volunteers gave written informed consent before beginning the testing and received monetary compensation for taking part in the study.

### Contingency degradation task

Habitual versus goal-directed behaviour was measured using a contingency degradation task used by Ersche et al., which consisted of 8 blocks of 120 trials, lasting 1 s each^31^. Figure 3a depicts the stimuli used in this task. Participants were presented with a white vase on the screen which could be filled with flowers every time the space bar key was pressed. In 60% of the trials, the key press was associated with a financial reward of 20 pence and the message “YOU WIN” on the screen for 500 ms. In the first 3 blocks, the association between the action of pressing the key, and winning 20 pence was established (non-degraded action-outcome contingency). This duration has been shown to be enough to induce habits in humans^88^. In block 4, in addition to the original probability of 60%, participants also received a free reward with a 30% probability (partially degraded condition). In block 5, the chance of receiving the free reward was also 60%, which was equal to the probability of reward after pressing the key/performing an action (fully degraded condition). In the final 3 blocks, the initial contingencies were reinstated (non-degraded conditions), followed by a partially degraded condition in block 7, and a fully degraded condition in block 8. Table 4 depicts an overview of all blocks and conditions.

**Table 4:** Overview of the contingency degradation experimental design

Block number	Condition	*P* (20p received following action)	*P* (20p received without action)	Δ*P* (programmed contingency)
1	Non-degraded	0.60	0.00	0.60
2	Non-degraded	0.60	0.00	0.60
3	Non-degraded	0.60	0.00	0.60
4	Partially-degraded	0.60	0.30	0.30
5	Fully-degraded	0.60	0.60	0.00
6	Non-degraded	0.60	0.00	0.60
7	Partially-degraded	0.60	0.30	0.30
8	Fully-degraded	0.60	0.60	0.00

*p* probability, Δ*P* delta/difference of the two contingencies.

To study habitual control, a habitual responding index was created by subtracting the responses during the non-degraded condition (probability of 0.60) from the fully degraded condition (probability of 0.30 or 0) with a lower probability of gaining rewards. The increasingly negative values for this index indicate a greater tendency towards goal-directed behaviour, whereas, less negative and positive values point towards a bias towards habitual behaviour. Note this index did not take into account overall individual variability in response output.

### MRS data acquisition

The proton magnetic resonance spectroscopy (^1^H MRS) took place at the Wolfson Brain Imaging Centre, University of Cambridge (United Kingdom). Participants underwent whole-brain T_1_-weighted MR and single-voxel proton MRS scans using a 7T Terra MRI (Siemens). The scanner was equipped with a Nova single-channel transmit, and 32-channel array head coil for signal reception (Nova Medical). T_1_-weighted MP2RAGE^89^ images were acquired to guide voxel placement and used in the analysis to perform tissue corrections (see below). The following specifications were used: echo time = 1.99 ms, repetition time = 4300 ms, inversion times (1^st^/2^nd^) = 840/2370 ms, flip angles = 5/6°, acceleration factor (A $\gg$ P) = 3, bandwidth = 250 Hz/px, voxel size = 0.75 mm. To increase the SNR and the amount of GM in each voxel, the spectra were measured bilaterally from one 12 × 20 × 33 mm^3^ voxel at the anterior cingulate cortex, and two 20 × 20 × 20 mm^3^ voxels at the supplementary motor area, and occipital cortex (Fig. 1a). All the voxels were located manually by the same researcher (M.B). Clear landmarks were used while placing the voxels to increase the reliability of the voxel placements across subjects. Supplementary Fig. S1 in Supplementary Information displays the landmarks used for placement of voxels.

After acquiring the MP2RAGE image, and placing the voxels, a short-echo-time semi-LASER^90,91^ sequence was used to acquire the spectra, collecting 64 repetitions with TR/TE of 5000/26 ms. This semi-LASER approach has recently been shown to have similar accuracy to MEGA-sLASER (an edited MRS sequence) for quantification of GABA in human brain at 7T^92^ and we expect similar performance for Glu and Gln. For each voxel, we applied FASTESTMAP^93^ for B_0_ shimming, and made an individual calibration for variable radio frequency pulses with optimised relaxation delay (VAPOR) water suppression^94^. The Brain Dot Engine software provided by Siemens was used to acquire the scans.

### MRS data preparation and analysis

Within subjects, the 64 individual spectra files were corrected for effects of eddy currents, frequency, and phase shifts using MRspa version v1.5 f (Dinesh Deelchand, University of Minnesota, www.cmrr.umn.edu/downloads/mrspa) and converted to one single averaged file. For the preprocessing step using the MRspa, the same researcher (MB) has paged through each transient/individual average file and dropped averages that were corrupted. Next, LCModel^95^ version 6.2-3 was used with an automated fitting routine, to quantify metabolites including GABA, Glu, Gln and NAA, relative to Cr + PCr (creatine plus phosphocreatine). Individual component fitted spectra for those metabolites between 0.5 and 4.2 ppm were extracted for quality inspection. The metabolites were quantified by reference to 8 spectra acquired without water suppression just before or after the 64 spectral repetitions, and using a simulated basis set that included experimentally acquired macromolecule spectra. Supplementary Table S2 shows the MRS checklist according to a recent consensus by Lin et al.^53^. At the pre-processing step of the MRS data using MRspa, individual average files that were corrupted were removed. In the OCD group the following averages were removed: within the OCC [1, 4, and 1] averages and within the SMA [2, 2, and 3] averages were removed for 3 subjects. In the HV group, only one subject had a single corrupted average file out of 64 for the OCC voxel. For another healthy participant, due to a data collection error, 54 averages were collected instead of 64.

A segmentation analysis was performed using SPM12 and the MP2RAGE images to extract tissue fractions for each subject for Grey Matter (GM), White Matter (WM) and Cerebrospinal fluid (CSF), and performed partial volume corrections within subjects according to Harris et al.^96^ for GABA, and Provencher^97^ for the rest of the metabolites. Lastly, in order to avoid exclusion of values that are disorder/group specific and can provide insight into the nature of a disorder, a straight cut-off score (which is usually used) is not recommended^80^. Instead, per metabolite and per group, the average and standard deviation were calculated for Cramér-Rao Lower Bound of each metabolite, and individual metabolite concentrations. Next, values larger than 2SD from each group’s mean CRLB were excluded for both measures. The latter were according to Frangou et al.^98^. According to these criteria, the following data were excluded: within the SMA voxel, GABA in 1 healthy and 1 OCD subjects, and Gln in 2 participants with OCD, within the OCC voxel, 2 Glu and Gln in 2 healthy subjects, and 1 GABA in a healthy subject, and 3 GABA in patients. Lastly, 1 ACC and 1 OCC voxel were excluded for one OCD patient due to an error during data collection.

### Statistical analysis

An independent sample *t*-test was used for descriptive data, clinical measures and task performance. One-sided independent sample *t*-tests were used to study the differences in the neurometabolites such as the Glu, GABA and Gln, and the clinical measures between the two groups. Whereas, to explore the differences in quality measure variables such as the CRLB, FWHM, brain tissues, and the rest of the neurometabolites, a two-sided independent sample *t*-test was used. When the normality condition was not satisfied using a Shapiro–Wilk test, the Mann–Whitney U test was used instead. Additionally, an analysis of covariance (ANCOVA) was performed using NAA levels as covariate to control for differences in neuronal integrity. For correlational analysis, Pearson or Spearman rank correlations were used depending on the data distribution. In addition to *p*-values, the *p*-values corrected for False Discovery Rates (FDR) are reported according to the correction suggested by Benjamini & Hochberg^99^ for each section of the results, with *p* < 0.05. Additionally, a hierarchical linear regression (HRL) model was built to predict Glu levels from GABA concentrations while controlling for Group and Voxel locations. As GABA values were not normally distributed, a boxcox transformation was performed to centralise the values and remove the skewness. The source code used for the FDR correction can be found on GitHub: https://github.com/carbocation/falsediscovery. The R code for HRL model is shared in Supplementary Information including the formula used for *boxcox* transformation of GABA values. Rstudio Version 3.0, Built 554 was used. SPSS version 28 was used for behavioural analysis of contingency degradation data and the ANCOVA. MATLAB R2018a was used to extract the tissue fractions within each voxel. The rest of the analysis was performed using Python version 3.7.6.

### Reporting summary

Further information on research design is available in the Nature Portfolio Reporting Summary linked to this article.

## Supplementary information

Supplementary Information

Peer Review File

Reporting Summary

## Data Availability

The data generated in this study are provided in the Source Data file which can be accessed here: 10.5281/zenodo.7832197. The raw MRS data can also be accessed using the same link, including the simulated basis set that was used during the data analysis. However, the structural images will be provided upon request and with restricted access using the following link: 10.5281/zenodo.7812441. The latter is due to the inability to fully anonymise these data. Source data are provided with this paper.

## Source data

Source Data

## Code availability

The R code for the Hierarchical Linear Regression, and the MATLAB code to extract the grey matter, white matter and CSF tissue fractions within individual voxels can be accessed here: 10.5281/zenodo.7832197.

## References

[1] Robbins TW, Gillan CM, Smith DG, de Wit S, Ersche KD (2012). Neurocognitive endophenotypes of impulsivity and compulsivity: towards dimensional psychiatry. Trends Cogn. Sci..

[2] Ziegler G (2019). Compulsivity and impulsivity traits linked to attenuated developmental frontostriatal myelination trajectories. Nat. Neurosci..

[3] Everitt, B. J. & Robbins, T. W. Drug addiction: Updating actions to compulsions to habits ten years on. *Annual Review of Psychology* **67**, 23–50 (2016). 10.1146/annurev-psych-122414-033457 26253543

[4] Saxena S, Rauch SL, Saxena S, Rauch SL (2000). Functional neuroimaging and the neuroanatomy of obsessive-compulsive disorder. Psychiatr. Clin. North Am..

[5] Bijanki KR (2021). Defining functional brain networks underlying obsessive–compulsive disorder (OCD) using treatment-induced neuroimaging changes: a systematic review of the literature. J. Neurol. Neurosurg. Psychiatry.

[6] de Wit SJ (2012). Presupplementary motor area hyperactivity during response inhibition: a candidate endophenotype of obsessive-compulsive disorder. Am. J. Psychiatry.

[7] Chakrabarty K, Bhattacharyya S, Christopher R, Khanna S (2005). Glutamatergic dysfunction in OCD. Neuropsychopharmacology.

[8] Graat, I., Figee, M. & Denys, D. Neurotransmitter dysregulation in OCD. In *Obsessive-compulsive Disorder: Phenomenology, Pathophysiology, and Treatment*. Vol. 25, 271–274 (Oxford University Press, 2017).

[9] Pittenger, C. The pharmacological treatment of refractory OCD. In *Obsessive-Compulsive Disorder: Phenomenology, Pathophysiology, and Treatment*. (ed. Pittenger, C.) (Oxford University Press, 2017).

[10] Brennan BP, Rauch SL, Jensen JE, Pope HG (2013). A critical review of magnetic resonance spectroscopy studies of obsessive-compulsive disorder. Biol. Psychiatry.

[11] Biria M, Cantonas LM, Banca P (2021). Magnetic Resonance Spectroscopy (MRS) and Positron Emission Tomography (PET) Imaging in Obsessive-Compulsive Disorder. Curr. Top. Behav. Neurosci..

[12] Simpson HB (2012). Investigation of cortical glutamate–glutamine and γ-aminobutyric acid in obsessive–compulsive disorder by proton magnetic resonance spectroscopy. Neuropsychopharmacology.

[13] Li Y (2019). Investigation of anterior cingulate cortex gamma-aminobutyric acid and glutamate-glutamine levels in obsessive-compulsive disorder using magnetic resonance spectroscopy. BMC Psychiatry.

[14] Carrasco M (2013). Increased error‐related brain activity in youth with obsessive‐compulsive disorder and unaffected siblings. Depress. Anxiety.

[15] Endrass T, Klawohn J, Schuster F, Kathmann N (2008). Overactive performance monitoring in obsessive-compulsive disorder: ERP evidence from correct and erroneous reactions. Neuropsychologia.

[16] Alexander WH, Brown JW (2011). Medial prefrontal cortex as an action-outcome predictor. Nat. Neurosci..

[17] Kolling N, Behrens TEJ, Wittmann MK, Rushworth MFS (2016). Multiple signals in anterior cingulate cortex. Curr. Opin. Neurobiol..

[18] Fitzgerald KD (2005). Error-related hyperactivity of the anterior cingulate cortex in obsessive-compulsive disorder. Biol. psychiatry.

[19] Hauser TU (2017). Increased fronto-striatal reward prediction errors moderate decision making in obsessive–compulsive disorder. Psychol. Med..

[20] Murray GK (2019). Dopaminergic drug treatment remediates exaggerated cingulate prediction error responses in obsessive-compulsive disorder. Psychopharmacology.

[21] Norman LJ (2019). Error processing and inhibitory control in obsessive-compulsive disorder: a meta-analysis using statistical parametric maps. Biol. Psychiatry.

[22] Alexander GE, Delong MR, Strick PL (1986). Parallel organization of functionally segregated circuits linking basal ganglia and cortex. Annu. Rev. Neurosci..

[23] Balleine BW, O’Doherty JP (2010). Human and rodent homologies in action control: corticostriatal determinants of goal-directed and habitual action. Neuropsychopharmacology.

[24] Guida, P. et al. An fMRI meta-analysis of the role of the striatum in everyday-life vs laboratory-developed habits. *Neurosci. Biobehav. Rev.* **141**, 104826 (2022). 10.1016/j.neubiorev.2022.104826 35963543

[25] Seger CA (2018). Corticostriatal foundations of habits. Curr. Opin. Behav. Sci..

[26] D’Urso G (2016). Transcranial direct current stimulation for obsessive–compulsive disorder: a randomized, controlled, partial crossover trial. Depress. Anxiety.

[27] Gowda SM (2019). Efficacy of pre-supplementary motor area transcranial direct current stimulation for treatment resistant obsessive compulsive disorder: a randomized, double blinded, sham controlled trial. Brain Stimul..

[28] Moffett JR, Ross B, Arun P, Madhavarao CN, Namboodiri AMA (2007). N-Acetylaspartate in the CNS: from neurodiagnostics to neurobiology. Prog. Neurobiol..

[29] Foa EB, Kozak MJ, Salkovskis PM, Coles ME, Amir N (1998). The validation of a new obsessive-compulsive disorder scale: the obsessive-compulsive inventory. Psychol. Assess..

[30] Goodman WK (1989). The Yale-Brown obsessive compulsive scale: I. Development, use, and reliability. Arch. Gen. Psychiatry.

[31] Ersche KD (2021). Reduced Glutamate Turnover in the putamen is linked with automatic habits in human cocaine addiction. Biol. Psychiatry.

[32] Gillan CM, Robbins TW, Sahakian BJ, van den Heuvel OA, van Wingen G (2016). The role of habit in compulsivity. Eur. Neuropsychopharmacology.

[33] O’Neill J (2016). Cingulate and thalamic metabolites in obsessive-compulsive disorder. Psychiatry Res Neuroimaging.

[34] de Salles Andrade JB (2019). An MRI study of the metabolic and structural abnormalities in obsessive-compulsive disorder. Front. Hum. Neurosci..

[35] Naaijen J (2016). Fronto-striatal glutamate in autism spectrum disorder and obsessive compulsive disorder. Neuropsychopharmacology.

[36] Starck G (2008). A 1H magnetic resonance spectroscopy study in adults with obsessive compulsive disorder: Relationship between metabolite concentrations and symptom severity. J. Neural Transm..

[37] Brennan BP (2015). An examination of rostral anterior cingulate cortex function and neurochemistry in obsessive–compulsive disorder. Neuropsychopharmacology.

[38] Zhang Z (2016). Brain Gamma-Aminobutyric Acid (GABA) concentration of the prefrontal lobe in unmedicated patients with obsessive-compulsive disorder: a research of magnetic resonance spectroscopy. Shanghai Arch. Psychiatry.

[39] Zheng H (2020). Reduced anterior cingulate glutamate of comorbid skin-picking disorder in adults with obsessive-compulsive disorder. J. Affect Disord..

[40] Batistuzzo MC (2021). Lower ventromedial prefrontal cortex glutamate levels in patients with obsessive–compulsive disorder. Front Psychiatry.

[41] Yücel M (2008). Anterior cingulate glutamate-glutamine levels predict symptom severity in women with obsessive-compulsive disorder. Aust. N.Z. J. Psychiatry.

[42] Reggente N (2018). Multivariate resting-state functional connectivity predicts response to cognitive behavioral therapy in obsessive–compulsive disorder. Proc. Natl Acad. Sci..

[43] Stern ER (2017). Switching between internally and externally focused attention in obsessive-compulsive disorder: abnormal visual cortex activation and connectivity. Psychiatry Res Neuroimaging.

[44] Geffen T (2022). Functional connectivity alterations between default mode network and occipital cortex in patients with obsessive-compulsive disorder (OCD). Neuroimage Clin..

[45] Hou JM (2014). Resting-state functional connectivity abnormalities in patients with obsessive–compulsive disorder and their healthy first-degree relatives. J. Psychiatry Neurosci..

[46] Gonçalves ÓF (2015). Brain activation of the defensive and appetitive survival systems in obsessive compulsive disorder. Brain Imaging Behav..

[47] Ravindran A (2020). Functional connectivity in obsessive-compulsive disorder and its subtypes. Psychol. Med..

[48] Tadayonnejad R (2017). Pregenual anterior cingulate dysfunction associated with depression in OCD: an integrated multimodal fMRI/1H MRS study. Neuropsychopharmacology.

[49] Yücel M (2007). Functional and biochemical alterations of the medial frontal cortex in obsessive-compulsive disorder. Arch. Gen. Psychiatry.

[50] Tükel R, Aydın K, Ertekin E, Özyıldırım SŞ, Barburoğlu M (2015). 1H-magnetic resonance spectroscopy in obsessive–compulsive disorder: effects of 12 weeks of sertraline treatment on brain metabolites. Eur. Arch. Psychiatry Clin. Neurosci..

[51] Tükel R, Aydin K, Ertekin E, Özyildirim SŞ, Taravari V (2014). Proton magnetic resonance spectroscopy in obsessive–compulsive disorder: Evidence for reduced neuronal integrity in the anterior cingulate. Psychiatry Res Neuroimaging.

[52] Joon Hwan Jang MD (2006). A proton MRSI study of brain N-acetylaspartate level after 12 weeks of citalopram treatment in drug-naive patients with obsessive-compulsive disorder. Am. J. Psychiatry.

[53] Lin, A. et al. Minimum reporting standards for in vivo magnetic resonance spectroscopy (MRSinMRS): experts’ consensus recommendations. *NMR Biomed.* **34**, e4484 (2021). 10.1002/nbm.4484 PMC8647919 33559967

[54] Steel A, Mikkelsen M, Edden RAE, Robertson CE (2020). Regional balance between glutamate+glutamine and GABA+ in the resting human brain. Neuroimage.

[55] Sukenik N (2021). Neuronal circuits overcome imbalance in excitation and inhibition by adjusting connection numbers. Proc. Natl Acad. Sci..

[56] Rideaux R (2021). No balance between glutamate+glutamine and GABA+ in visual or motor cortices of the human brain: a magnetic resonance spectroscopy study. Neuroimage.

[57] Nguyen VT, Breakspear M, Cunnington R (2014). Reciprocal interactions of the SMA and cingulate cortex sustain premovement activity for voluntary actions. J. Neurosci..

[58] Apergis-Schoute AM (2018). Hyperconnectivity of the ventromedial prefrontal cortex in obsessive-compulsive disorder. Brain Neurosci. Adv..

[59] Soriano-Mas, C., & Harrison, B. J. Brain functional connectivity in Obsessive-Compulsive disorder. *In Obsessive Compulsive Disorder: Phenomenology, Pathophysiology, and Treatment* (Oxford University Press, 2017).

[60] Gillan CM, Robbins TW (2014). Goal-directed learning and obsessive–compulsive disorder. Philos. Trans. R. Soc. B Biol. Sci..

[61] Vaghi MM (2019). Action-outcome knowledge dissociates from behavior in obsessive-compulsive disorder following contingency degradation. Biol. Psychiatry Cogn. Neurosci. Neuroimaging.

[62] Duan LY (2021). Controlling one’s world: Identification of sub-regions of primate PFC underlying goal-directed behavior. Neuron.

[63] de Wit S (2012). Corticostriatal connectivity underlies individual differences in the balance between habitual and goal-directed action control. J. Neurosci..

[64] Zwosta K, Ruge H, Goschke T, Wolfensteller U (2018). Habit strength is predicted by activity dynamics in goal-directed brain systems during training. Neuroimage.

[65] Tanaka SC, Balleine BW, O’Doherty JP (2008). Calculating consequences: brain systems that encode the causal effects of actions. J. Neurosci..

[66] Liljeholm M, Tricomi E, O’Doherty JP, Balleine BW (2011). Neural correlates of instrumental contingency learning: differential effects of action–reward conjunction and disjunction. J. Neurosci..

[67] Aron AR, Robbins TW, Poldrack RA (2014). Inhibition and the right inferior frontal cortex: one decade on. Trends Cogn. Sci..

[68] Rae CL, Hughes LE, Anderson MC, Rowe XB (2015). The prefrontal cortex achieves inhibitory control by facilitating subcortical motor pathway connectivity. J. Neurosci..

[69] Tomiyama H (2022). Increased functional connectivity between presupplementary motor area and inferior frontal gyrus associated with the ability of motor response inhibition in obsessive–compulsive disorder. Hum. Brain Mapp..

[70] Mostofsky SH, Simmonds DJ (2008). Response inhibition and response selection: two sides of the same coin. J. Cogn. Neurosci..

[71] Sumner P (2007). Human medial frontal cortex mediates unconscious inhibition of voluntary action. Neuron.

[72] Dayan A. (2017). Enhanced action tendencies in obsessive-compulsive disorder: an ERP study. Behav. Res. Ther..

[73] Vassena E (2020). Surprise, value and control in anterior cingulate cortex during speeded decision-making. Nat. Hum. Behav..

[74] Botvinick MM, Cohen JD, Carter CS (2004). Conflict monitoring and anterior cingulate cortex: an update. Trends Cogn. Sci..

[75] Botvinick M, Nystrom LE, Fissell K, Carter CS, Cohen JD (1999). Conflict monitoring versus selection-for-action in anterior cingulate cortex. Nature.

[76] Botvinick MM, Carter CS, Braver TS, Barch DM, Cohen JD (2001). Conflict monitoring and cognitive control. Psychol. Rev..

[77] Riesel A, Kathmann N, Endrass T (2014). Overactive performance monitoring in obsessive–compulsive disorder is independent of symptom expression. Eur. Arch. Psychiatry Clin. Neurosci..

[78] Gehring WJ, Goss B, Coles MGH, Meyer DE, Donchin E (2016). A neural system for error detection and compensation. Psychological Sci..

[79] Musazzi L, Treccani G, Mallei A, Popoli M (2013). The action of antidepressants on the glutamate system: regulation of glutamate release and glutamate receptors. Biol. Psychiatry.

[80] Kreis R (2016). The trouble with quality filtering based on relative Cramér-Rao lower bounds. Magn. Reson Med..

[81] Rodriguez CI (2015). In vivo effects of ketamine on glutamate-glutamine and gamma-aminobutyric acid in obsessive-compulsive disorder: Proof of concept. Psychiatry Res Neuroimaging.

[82] Montgomery SA, Asberg M (1979). A new depression scale designed to be sensitive to change. Br. J. Psychiatry.

[83] Spielberger, C. D., Gorsuch, R. L., Lushene, R., Vagg, P. R. & Jacobs, G. A. *Manual for the state-trait anxiety inventory* (Consulting Psychologists Press, 1983).

[84] Nelson, H. E. National Adult Reading Test (NART): Test Manual. NFER, Windsor: Nelson (1982).

[85] Lewin AB (2011). Refining clinical judgment of treatment outcome in obsessive–compulsive disorder. Psychiatry Res..

[86] Buhr K, Dugas MJ (2002). The intolerance of uncertainty scale: psychometric properties of the English version. Behav. Res. Ther..

[87] Ramakrishnan S, Robbins TW, Zmigrod L (2022). The Habitual Tendencies Questionnaire: a tool for psychometric individual differences research. Personal. Ment. Health.

[88] de Wit S (2018). Shifting the balance between goals and habits: five failures in experimental habit induction. J. Exp. Psychol. Gen..

[89] Marques JP (2010). MP2RAGE, a self bias-field corrected sequence for improved segmentation and T1-mapping at high field. Neuroimage.

[90] Deelchand DK (2015). Two-site reproducibility of cerebellar and brainstem neurochemical profiles with short-echo, single-voxel MRS at 3T. Magn. Reson. Med..

[91] Öz G, Tkáč I (2011). Short-echo, single-shot, full-intensity proton magnetic resonance spectroscopy for neurochemical profiling at 4 T: validation in the cerebellum and brainstem. Magn. Reson. Med..

[92] Hong D, Rankouhi SR, Thielen JW, van Asten JJA, Norris DG (2019). A comparison of sLASER and MEGA-sLASER using simultaneous interleaved acquisition for measuring GABA in the human brain at 7T. PLoS One.

[93] Gruetter R, Tkáč I (2000). Field mapping without reference scan using asymmetric echo‐planar techniques. Magn. Reson. Med..

[94] Tkáč I, Starčuk Z, Choi I-Y, Gruetter R (1999). In vivo 1H NMR spectroscopy of rat brain at 1 ms echo time. Magn. Reson. Med..

[95] Provencher SW (1993). Estimation of metabolite concentrations from localized in vivo proton NMR spectra. Magn. Reson. Med..

[96] Harris AD, Puts NAJ, Edden RAE (2015). Tissue correction for GABA-edited MRS: considerations of voxel composition, tissue segmentation, and tissue relaxations. J. Magn. Reson Imaging.

[97] Provencher, S. W. LCModel Manual [Online]. Available at: http://s-provencher.com/pub/LCModel/manual/manual.pdf (Accessed 21 April 2021).

[98] Frangou P (2019). Learning to optimize perceptual decisions through suppressive interactions in the human brain. Nat. Commun..

[99] Benjamini Y, Hochberg Y (1995). Controlling the false discovery rate: a practical and powerful approach to multiple testing. J. R. Stat. Soc. Ser. B (Methodol.).

