# Supplementary material for: Cortical glutamate and GABA are related to compulsive behaviour in individuals with obsessive compulsive disorder and healthy controls
Source: Nat Commun. 2023 Jun 27;14:3324. doi: 10.1038/s41467-023-38695-z (PMC10300066; doi:10.1038/s41467-023-38695-z)
Supplement: Supplementary file 1 — Supplementary Information [file 41467_2023_38695_MOESM1_ESM.pdf]

Supplementary Information

Cortical glutamate and GABA are related to compulsive behaviour in individuals with obsessive compulsive disorder and healthy controls

Marjan Biria, Paula Banca, Máiréad P. Healy*, Engin Keser*, Stephen J. Sawiak, Christopher T Rodgers, Catarina Rua, Ana Maria Frota Lisbôa Pereira de Souza, Aleya A.Marzuki, Akeem Sule, Karen D. Ersche, Trevor W. Robbins

* These authors had equal contribution

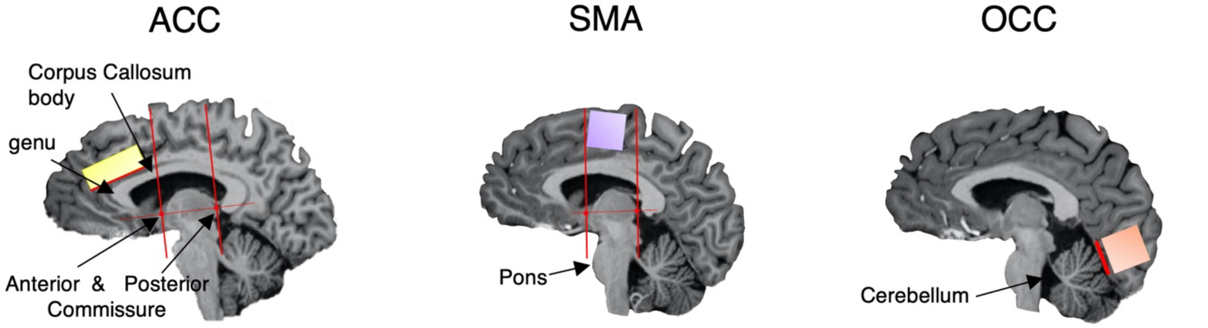

Figure S1 shows the location of the three voxels in a sagittal space, while indicating the landmarks used to increase their consistent placement and to avoid an overlap between the ACC and SMA voxels. First, a horizontal line was drawn between the anterior and posterior commissure with two vertical lines going through each of them perpendicularly. These lines are depicted in red for the ACC and SMA voxels. The ACC box (in yellow) was placed in front of the line going through the anterior commissure with the outer left corner of the box being in front of the genu of corpus callosum. The SMA box (in purple) was placed above the pons and between the two red lines. The upper side of the box was placed parallel with the skull above it. Lastly, the OCC box (in orange) was placed in the outermost corner of the occipital lobes while avoiding the skull and sinuses, with the lower side of the box being parallel with the red line above cerebellum. All three voxels were placed bilaterally and the SMA box included regions from supplementary and pre-supplementary motor areas. Acronyms: ACC = anterior cingulate cortex, SMA = supplementary motor area, OCC = occipital cortex.

Table S1 Measures of magnetic resonance spectroscopy quality and tissue composition.

Measures	HV M ± SD	OCD M ± SD	t / U	DF	*p*	*d*	95% CI
**ACC**							
SNR	56.47 ± 6.25	60.97 ± 6.29	2.77	58	0.007	0.71	[1.26, 7.74]
FWHM	0.02 ± 0.00	0.020 ± 0.00	365^{U}	NA	0.16	$-0.03^{\eta p2}$	[-0.005, 0.0]
GM	0.84 ± 0.03	.84 ± .04	0.18	58	0.86	0.04	[-0.015, 0.018]
WM	0.10 ± 0.03	0.10 ± 0.02	- 0.20	58	0.84	-0.05	[-0.014, 0.011]
% CRLB GABA	9.17 ± 1.28	9.20 ± 1.18	471^{U}	NA	0.74	$-0.002^{\eta p2}$	[-1.0, 1.0]
% CRLB Glu	1.90 ± 0.30	1.83 ± 0.38	420^{U}	NA	0.45	$-0.009^{\eta p2}$	[0.0, 0.0]
% CRLB Gln	6.80 ± 1.00	6.27 ± 1.23	331^{U}	NA	0.07	$-0.05^{\eta p2}$	[-1.0, 0.0]
% CRLB NAA	1.00 ± 0.00	1.00 ± 0.00	NA	NA	NA	NA	NA
**SMA**							
SNR	57.27 ± 10.30	54.74 ± 13.51	- 0.819	59	0.41	-0.21	[-8.67, 3.62]
FWHM	0.025 ± 0.00	0.03 ± 0.01	501^{U}	NA	0.57	$-0.005^{\eta p2}$	[0.0, 0.0]
GM	0.71 ± 0.06	0.07 ± 0.50	0.435	59	0.66	0.11	[-0.023, 0.036]
WM	0.17 ± 0.04	0.16 ± 0.04	- 0.618	59	0.54	-0.16	[-0.026, 0.014]
% CRLB GABA	9.80 ± 2.91	9.55 ± 2.01	482^{U}	NA	0.46	$-0.001^{\eta p2}$	[-1.0, 1.0]
% CRLB Glu	2.07 ± 0.25	2.19 ± 0.75	483^{U}	NA	0.68	$-0.003^{\eta p2}$	[0, 0]
% CRLB Gln	12.43 ± 6.69	12.10 ± 5.39	461^{U}	NA	0.69	$-0.002^{\eta p2}$	[-2, 3]
% CRLB NAA	1.07 ± 0.36	1.06 ± 0.25	478^{U}	NA	0.61	$-0.004^{\eta p2}$	[0, 0]
**OCC**							
SNR	80.50 ± 12.47	78.23 ± 18.79	- 0.555	59	0.58	-0.14	[-10.47, 5.92]
FWHM	0.03 ± 0.00	0.03 ± 0.00	0.500	59	0.62	0.13	[-0.001, 0.002]
GM	0.80 ± 0.03	0.81 ± 0.04	1.12	58	0.26	0.29	[-0.008, 0.029]
WM	0.16 ± 0.03	0.15 ± 0.03	- 0.882	58	0.38	-0.23	[-0.024, 0.009]
% CRLB GABA	9.20 ± 2.10	10.53 ± 6.70	359^{U}	NA	0.60	$-0.0001^{\eta p2}$	[-2.0, 1.0]
% CRLB Glu	1.87 ± 0.43	1.79 ± 0.56	381^{U}	NA	0.45	$-0.007^{\eta p2}$	[0, 0]
% CRLB Gln	4.73 ± 1.78	4.97 ± 1.45	504^{U}	NA	0.15	$-0.0144^{\eta p2}$	[0, 0]
% CRLB NAA	1.07 ± 0.25	1.14 ± 0.35	467^{U}	NA	0.76	$-0.0141^{\eta p2}$	[0, 0]

The data for this table are provided in the Source Data file. All tests were two-sided. For the ACC voxel the sample size for Glu and GABA in the OCD group was (n = 30) and in HV (n =30). For the SMA voxel the sample size for Glu in the OCD group was (n = 31) and in the HV it was (n = 30), for GABA in the OCD group it was (n = 30) and in the HV the sample size was (n = 29). Lastly, for the OCC voxel the sample size for Glu in the OCD group was (n = 30) and in the HV it was (n = 28), for GABA in the OCD group it was (n = 27) and in the HV the sample size was (n = 29). Acronyms: ACC = anterior cingulate cortex, SMA = supplementary motor area, OCC = occipital cortex, SNR: signal-to-noise ratio, FWHM = full width at half maximum in ppm units, GM = gray matter fraction, WM = white matter fraction, % CRLB: percentage Cramer-Rao Lower Bound, GABA = γ-amino-butyric acid, Glu = glutamate, Gln = Glutamine, NAA = N-acetylaspartate, *t* = independent sample t-test, *U* = Mann-Whitney *U* test, *M* = mean, SD = standard deviation, η_p^2 = partial eta-square (a measure of effect size for the U test), t = independent sample t-test, DF = degree of freedom, d = Cohen's d, CI = Confidence Interval of the t-test, NA = not applicable, test could not be performed as values for all subject for both groups were the same, HV = healthy volunteers, OCD = obsessive-compulsive disorder.

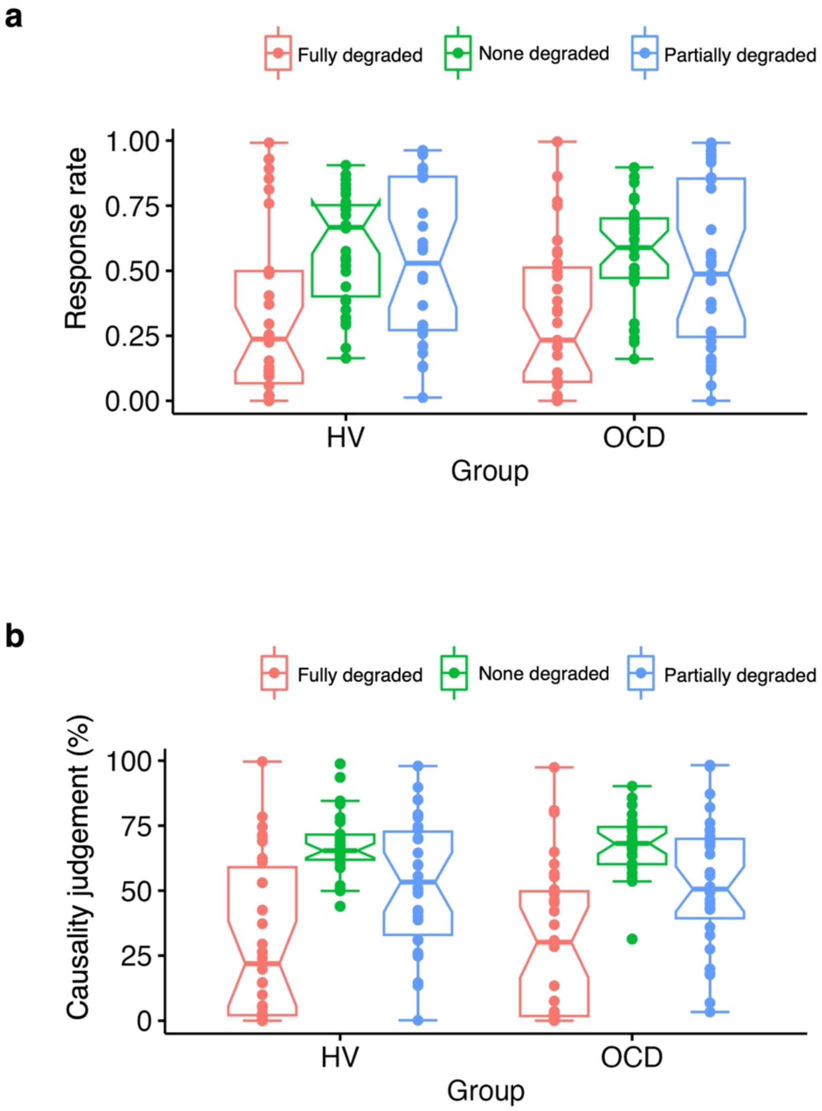

Figure S2 shows the habitual responding and the corresponding subjective causality judgements in OCD (n = 31) and healthy subjects (n = 30) between conditions of fully degraded (orange), none-degraded (green), and partially-degraded (blue). A mixed repeated measure ANOVA was performed to study the effects of the 3 conditions on response rate and causality judgement. **(a)** displays the response rates per second, with only a significant condition effect ($F(1,59) = 6.61$, $p = 0.01$, $\eta_p^2 = 0.10$, 95% CI[0.42, 0.54]), **(b)** shows the subjective causality judgements in percentage for OCD and health volunteers, again there was only a significant condition effect ($F(1,59) = 93.84$, $p < 0.001$, $\eta_p^2 = 0.61$, CI[45.53, 54.69]). The filled circles show the individual data points, the boxes starts from the first to the third quartile with a horizontal line and a notch through the median. The whiskers go from each quartile to minimum and maximum. The notch approximates a 95% confidence interval for the median. If the notches of two boxes do not overlap, this suggests that the medians are significantly different. The points outside whiskers represent the outliers. The data for this figure are provided in the Source Data file. Acronyms: HV = healthy volunteers, OCD = obsessive-compulsive disorder.

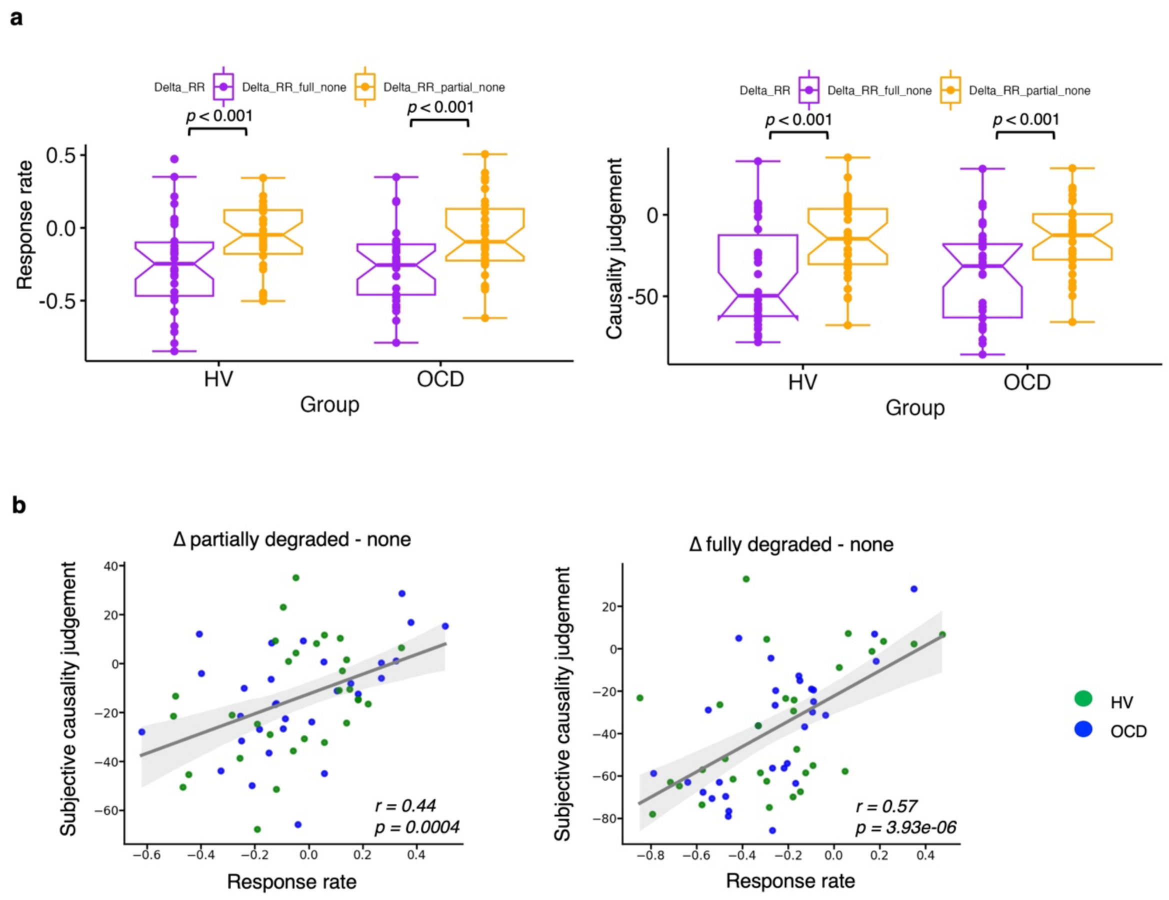

Figure S3 (a) shows the results of a mixed repeated measure ANOVA for the habitual responding on the left and the corresponding subjective causality judgements on the right in OCD (n = 31) and healthy subjects (n = 30). There are two conditions of fully minus none, and partially minus none degradation. The habitual responding index used for the main analysis is represented in yellow in the response rate plot on the left. Although both groups showed significant differences between conditions for response rate (F(1,59) = 30.70, *p* = 0.000, ηp2= 0.32, 95% CI[-0.21, -0.95]) and causality judgement (F(1,59) = 48.53, *p* = 0.000, η_p^2= 0.45, 95% CI[-0.32, -0.20]), no interaction with group was found. The filled circles show the individual data points, the boxes starts from the first to the third quartile with a horizontal line and a notch through the median. The whiskers go from each quartile to minimum and maximum. The notch approximates a 95% confidence interval for the median. If the notches of two boxes do not overlap, this suggests that the medians are significantly different. The points outside whiskers represent the outliers. **(b)** displays the significant relationships between the habitual responding and the corresponding subjective causality judgements for both groups with the fitted line for the entire sample in gray, between the conditions of none minus partial, and none minus full degradation, respectively. The data for this figure are provided in the Source Data file. Acronyms: HV = healthy volunteers, OCD = obsessive-compulsive disorder, *r* = Pearson's r correlation coefficient, η_p^2 = partial eta-square as a measure of effect size.

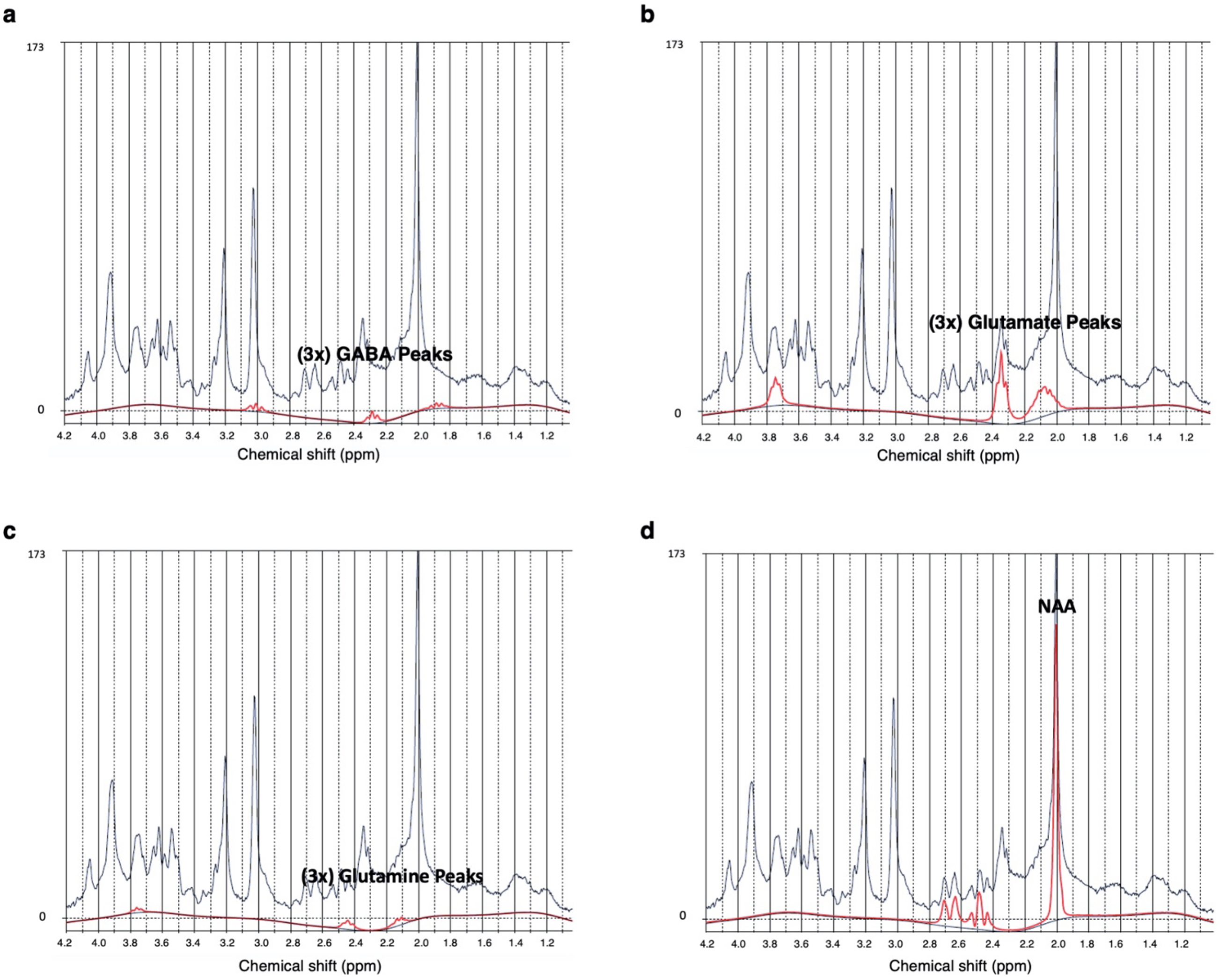

Figure S4 shows examples of the LCModel analysis of in vivo ^{1}H MR spectra acquired from a healthy participant at 7T (semi-LASER, echo time/repetition time = 1.99/4300 ms, from a 20 x 20 x 20mm voxel placed bilaterally at supplementary motor area). The x-axis depicts the chemical shift, the acquired spectrum is plotted in black and the fit is presented in red for **(a)** GABA, **(b)** Glutamate, **(c)** Glutamine, and **(d)** NAA. Acronyms: GABA = γ-amino-butyric acid, NAA = N-acetylaspartate, ppm = parts per million.

Table S2 MRS checklist according to Lin et al. 2021[1]

**Site name:**	Wolfson Brain Imaging Centre, Department of Clinical Neurosciences, University of Cambridge
**1. Hardware**	
**a**. Field strength [T]:	7T
**b.** Manufacturer:	Siemens
**c.** Model (software version if available)	Magnetom 7T Terra
**d.** RF coils: nuclei (transmit/receive), number of channels, type, body part	Nova 1Tx32Rx 1H head coil
**e.** Additional hardware (eg shim inserts, dielectric pads)	None
**2. Acquisition**	
**a.** Pulse sequence	semi-LASER
**b.** Volume of Interest (VOI) locations	bilaterally at anterior cingulate cortex (ACC), supplementary (including pre-supplementary) motor area (SMA), occipital cortex (OCC), see Fig.S1 above.
**c.** Nominal VOI size [cm^3, mm^3]	ACC: 12 x 20 x 33 cm3, SMA: 2 x 2 x 2 cm3, OCC: 2 x 2 x 2cm3
**d.** Repetition Time (TR), Echo Time (TE)	TR = 5000, total TE = 26ms, TE 1, 2, 3 = 7, 10, 9ms
**e.** Total number of excitations or acquisitions per spectrum in time series for kinetic studies<br>i. Number of Averaged spectra per time-point<br>ii. Averaging method (e.g. block-wise or moving average)<br>iii. Total number of spectra (acquired / in time-series)	64 averages with water-suppression plus spectra without water suppression, and 8 water peak files were collected (4 at the beginning and 4 at the end).
**f.** Additional sequence parameters (spectral width in Hz, number of spectral points, frequency offsets)	The standard CMRR short-TE sLASER protocol were followed.
**g.** Water Suppression Method	VAPOR
**h.** Shimming Method, reference peak, and thresholds for "acceptance of shim" chosen	FASTESTMAP, acceptable shims have linewidth <= 15Hz otherwise manual shimming was performed.
**i.** Triggering or motion correction method (respiratory, peripheral, cardiac triggering, incl. device used and delays)	None
**3. Data analysis methods and outputs**	
**a.** Analysis software	LCModel version 6.2-3, Mrspa v1.5f (Dinesh Deelchand, University of Minnesota, www.cmrr.umn.edu/downloads/mrspa)
**b.** Processing steps deviating from quoted reference or product	No deviations took place in the pre-processing steps from Provencher (2021). At the level of the data analysis, a segmentation analysis was performed using SPM12 and the MP2RAGE images to extract tissue fractions for each subject and each individual voxel for gray matter, white matter and cerebrospinal fluid. Next, partial volume corrections were performed within subjects/voxels according to Harris et al., (2015) for GABA, and Provencher (2021) for the rest of the metabolites.
**c.** Output measure (e.g. absolute concentration, institutional units, ratio)	Ratio to total creatine (creatine + phosphocreatine).
**d.** Quantification references and assumptions, fitting model assumptions	The sead_7T_26ms_11Dec2013.BASIS' file supplied with MRspa was used. This basis set contains simulated spectra and a measured macromolecular baseline.

4. Data Quality	
**a.** Reported variables (SNR, Linewidth (with reference peaks))	Metabolite ratios relative to total creatine (creatine + phosphocreatine), SNR, FWHM, CRLB as reported in LCModel output file.
**b.** Data exclusion criteria	At the preprocessing step of the MRS data using MRspa, individual average files that were corrupted were removed. In the OCD group the following averages were removed: within the OCC [1, 4, and 1] averages and within the SMA [2, 2, and 3] averages were removed for 3 subjects. In the HV group, only one subject had a single corrupted average file out of 64 for the OCC voxel. For another healthy participant, due to a data collection error, 54 averages were collected instead of 64.<br><br>In order to avoid exclusion of values that are disorder/group specific and can provide insight into the nature of OCD, we followed Kreis (2016), and avoided using a straight cut-off score for CRLB. Instead, per metabolite and per group, the average and standard deviation were calculated for Cramér-Rao Lower Bound of each metabolite, and individual metabolite concentrations. Next, values larger than 2SD from each group's mean CRLB and concentration levels were excluded. The latter were according to Frangou et al. (2019). According to this criteria, the following data were excluded: within the SMA voxel, GABA in one healthy and one OCD subjects, and Gln in two OCD patients were excluded; within the OCC voxel, Glu and Gln in two healthy subjects, GABA in one healthy subject and three patients were excluded. One ACC and one OCC voxels were excluded for one OCD patient due to an error during data collection which led to loss of data.
**c.** Quality measures of postprocessing Model fitting	See above.
**d.** Sample Spectrum	Fig.S4 shows sample spectra for GABA, Glutamate, Glutamine and NAA peaks.

Hierarchical Linear Regression

A 6 stage hierarchical linear regression model was conducted with Glu levels as dependent variable. The boxcox transformed GABA levels were entered at stage 1 to predict Glu levels. The group was entered at stage 2, with an interaction term at stage 3, voxel was added at stage 4, an interaction term was added for group and voxel at stage 5, an interaction term was added for GABA and voxels at stage 6. At stage 1, the model revealed that GABA levels could significantly predict Glu levels ($F_{1, 171} = 48.51$, $p < 0.001$) and accounted for 21% of their variations. Introducing group in stage 2 explained an additional 1% of Glu level variations and this change in model was significant ($F_{2,170} = 25.84$, $p < 0.001$). An interaction term between GABA and group was added to the model at stage 3, adding an additional 3% in explaining the Glu variance which had a significant change in the model ($F_{3,169} = 19.04$, $p < 0.001$). There was an interaction between GABA levels and OCD group (t(58) = -2.10, p = 0.03). Adding voxels at stage 4 added around 41% in explained variance and a significant change in the model ($F_{5,167} = 65.10$, $p < 0.001$). At stage 5 an interaction term between groups and voxels was added and although the model was significant ($F_{11,161} = 32.00$, $p < 0.001$), this change did not add anything to the explained variation. Similarly, at the final stage, adding an interaction term between GABA and voxels did not contribute anything to the explained variance, while the model was still significant ($F_{7,165} = 48.90$, $p < 0.001$). Together all variables could explain around 65% variance in Glu levels. After performing an ANOVA the model built at stage 4 was the most optimal one ($F_{2,167} = 114.69$, $p < 0.001$). Although Group (OCD vs HV) did contribute to this regression model by increasing the explained variance in Glu levels, the impact of Voxel was larger. Adding Group at Step 2 and 3 (with an interaction term with GABA) accounted for an additional 2% in explaining the variance in Glu concentrations. Whereas, the addition of Voxel at step 4 of the model had the largest impact by increasing the R^2 value by 41%.

R Code:

```
Box Cox transformation in R
library(MASS)
x = df$GABA
b <- boxcox(lm(x ~ 1))
# Exact lambda
lambda <- b$x[which.max(b$y)]
lambda # = -0.1010101which is used in the next formula to transform GABA
df$GABA_transformed <- (x ^ lambda - 1) / lambda

> #hierarchical regression using base R function lm
> model1 <- lm(Glu~GABA_transformed,data=df)
> model2 <- lm(Glu~GABA_transformed + Group,data=df)
> model3 <- lm(Glu~GABA_transformed * Group,data=df)
> model4 <- lm(Glu~GABA_transformed * Group + Voxel,data=df)
> model5 <- lm(Glu~GABA_transformed * Group * Voxel,data=df)
> model6 <- lm(Glu~(GABA_transformed * Group) + (GABA_transformed *Voxel),data=df)

> summary(model1)

Call:
lm(formula = Glu ~ GABA_transformed, data = df)

Residuals:
    Min      1Q  Median      3Q     Max 
-3.6037 -0.8620  0.0019  0.9297  3.5471 

Coefficients:
                 Estimate Std. Error t value Pr(>|t|)    
(Intercept)        8.8567     0.4298  20.607  < 2e-16 ***
GABA_transformed   2.9869     0.4288   6.965 6.78e-11 ***
---
Signif. codes:  0 ‘***’ 0.001 ‘**’ 0.01 ‘*’ 0.05 ‘.’ 0.1 ‘ ’ 1

Residual standard error: 1.241 on 171 degrees of freedom
  (10 observations deleted due to missingness)
Multiple R-squared:  0.221,	Adjusted R-squared:  0.2164 
F-statistic: 48.51 on 1 and 171 DF,  p-value: 6.775e-11
```

```
> summary(model2)

Call:
lm(formula = Glu ~ GABA_transformed + Group, data = df)

Residuals:
    Min      1Q  Median      3Q     Max 
-3.7526 -0.8301  0.0341  0.8984  3.7013 

Coefficients:
                 Estimate Std. Error t value Pr(>|t|)    
(Intercept)        8.7075     0.4372  19.915  < 2e-16 ***
GABA_transformed   2.9810     0.4268   6.985 6.14e-11 ***
GroupOCD           0.3082     0.1878   1.641    0.103    
---
Signif. codes:  0 ‘***’ 0.001 ‘**’ 0.01 ‘*’ 0.05 ‘.’ 0.1 ‘ ’ 1

Residual standard error: 1.235 on 170 degrees of freedom
  (10 observations deleted due to missingness)
Multiple R-squared:  0.2331,	Adjusted R-squared:  0.2241 
F-statistic: 25.84 on 2 and 170 DF,  p-value: 1.588e-10

> summary(model3)

Call:
lm(formula = Glu ~ GABA_transformed * Group, data = df)

Residuals:
    Min      1Q  Median      3Q     Max 
-3.1619 -0.8336  0.0154  0.8380  3.8306 

Coefficients:
                          Estimate Std. Error t value Pr(>|t|)    
(Intercept)                 7.7686     0.6223  12.483  < 2e-16 ***
GABA_transformed            3.9432     0.6233   6.327 2.16e-09 ***
GroupOCD                    2.0487     0.8494   2.412   0.0169 *  
GABA_transformed:GroupOCD  -1.7805     0.8478  -2.100   0.0372 *  
---
Signif. codes:  0 ‘***’ 0.001 ‘**’ 0.01 ‘*’ 0.05 ‘.’ 0.1 ‘ ’ 1

Residual standard error: 1.223 on 169 degrees of freedom
  (10 observations deleted due to missingness)
Multiple R-squared:  0.2526,	Adjusted R-squared:  0.2394 
F-statistic: 19.04 on 3 and 169 DF,  p-value: 1.095e-10
> summary(model4)

Call:
lm(formula = Glu ~ GABA_transformed * Group + Voxel, data = df)

Residuals:
     Min       1Q   Median       3Q      Max 
-2.82170 -0.53384  0.03595  0.46649  2.88013 

Coefficients:
                          Estimate Std. Error t value Pr(>|t|)    
(Intercept)               10.48778    0.46333  22.636  < 2e-16 ***
GABA_transformed           1.82653    0.45578   4.007 9.23e-05 ***
GroupOCD                   1.33061    0.57819   2.301   0.0226 *  
VoxelOCC                  -2.09528    0.16039 -13.064  < 2e-16 ***
VoxelSMA                   0.01228    0.15938   0.077   0.9387    
GABA_transformed:GroupOCD -1.04722    0.57726  -1.814   0.0715 .  
---
Signif. codes:  0 ‘***’ 0.001 ‘**’ 0.01 ‘*’ 0.05 ‘.’ 0.1 ‘ ’ 1

Residual standard error: 0.8287 on 167 degrees of freedom
  (10 observations deleted due to missingness)
Multiple R-squared:  0.6609,	Adjusted R-squared:  0.6508 
F-statistic:  65.1 on 5 and 167 DF,  p-value: < 2.2e-16

> summary(model5)

Call:
lm(formula = Glu ~ GABA_transformed * Group * Voxel, data = df)

Residuals:
     Min       1Q   Median       3Q      Max 
-2.19396 -0.53615 -0.07111  0.46980  2.95951 

Coefficients:
                                   Estimate Std. Error t value Pr(>|t|)    
(Intercept)                         9.46827    1.07637   8.796 2.07e-15 ***
GABA_transformed                    2.77778    1.08076   2.570   0.0111 *  
GroupOCD                           -0.07551    1.44290  -0.052   0.9583    
VoxelOCC                           -0.63458    1.31569  -0.482   0.6302    
VoxelSMA                            0.76204    1.31384   0.580   0.5627    
GABA_transformed:GroupOCD           0.67248    1.48033   0.454   0.6502    
GABA_transformed:VoxelOCC          -1.34544    1.39954  -0.961   0.3378    
```

```
GABA_transformed:VoxelSMA          -0.72501    1.27268  -0.570   0.5697
GroupOCD:VoxelOCC                   0.96051    1.75675   0.547   0.5853
GroupOCD:VoxelSMA                   2.94335    1.77725   1.656   0.0996 .
GABA_transformed:GroupOCD:VoxelOCC -1.45033    1.87630  -0.773   0.4407
GABA_transformed:GroupOCD:VoxelSMA -3.05989    1.74120  -1.757   0.0808 .
---
Signif. codes:  0 ‘***’ 0.001 ‘**’ 0.01 ‘*’ 0.05 ‘.’ 0.1 ‘ ’ 1

Residual standard error: 0.812 on 161 degrees of freedom
  (10 observations deleted due to missingness)
Multiple R-squared:  0.6862,  Adjusted R-squared:  0.6647
F-statistic:    32 on 11 and 161 DF,  p-value: < 2.2e-16
```

```
> summary(model6)

Call:
lm(formula = Glu ~ (GABA_transformed * Group) + (GABA_transformed *
    Voxel), data = df)

Residuals:
     Min       1Q   Median       3Q      Max
-2.55220 -0.51679  0.02545  0.42433  2.76558

Coefficients:
                          Estimate Std. Error t value Pr(>|t|)
(Intercept)                 8.7996     0.7872  11.178  < 2e-16 ***
GABA_transformed            3.5621     0.7976   4.466 1.47e-05 ***
GroupOCD                    1.3530     0.5698   2.375   0.0187 *
VoxelOCC                   -0.1664     0.8729  -0.191   0.8490
VoxelSMA                    2.2358     0.8862   2.523   0.0126 *
GABA_transformed:GroupOCD  -1.0333     0.5689  -1.817   0.0711 .
GABA_transformed:VoxelOCC  -2.0386     0.9317  -2.188   0.0301 *
GABA_transformed:VoxelSMA  -2.2357     0.8686  -2.574   0.0109 *
---
Signif. codes:  0 ‘***’ 0.001 ‘**’ 0.01 ‘*’ 0.05 ‘.’ 0.1 ‘ ’ 1

Residual standard error: 0.8166 on 165 degrees of freedom
  (10 observations deleted due to missingness)
Multiple R-squared:  0.6747,  Adjusted R-squared:  0.6609
F-statistic:  48.9 on 7 and 165 DF,  p-value: < 2.2e-16
```

```
> anova(model1,model2,model3,model4,model5,model6)

Analysis of Variance Table

Model 1: Glu ~ GABA_transformed
Model 2: Glu ~ GABA_transformed + Group
Model 3: Glu ~ GABA_transformed * Group
Model 4: Glu ~ GABA_transformed * Group + Voxel
Model 5: Glu ~ GABA_transformed * Group * Voxel
Model 6: Glu ~ (GABA_transformed * Group) + (GABA_transformed * Voxel)
  Res.Df    RSS Df Sum of Sq        F    Pr(>F)
1    171 263.51
2    170 259.40  1     4.108   6.2308  0.013561 *
3    169 252.80  1     6.597  10.0055  0.001866 **
4    167 114.69  2   138.106 104.7341 < 2.2e-16 ***
5    161 106.15  6     8.545   2.1602  0.049502 *
6    165 110.03 -4    -3.876   1.4696  0.213839
---
Signif. codes:  0 ‘***’ 0.001 ‘**’ 0.01 ‘*’ 0.05 ‘.’ 0.1 ‘ ’ 1
```

Supplementary References

1. Lin, A. *et al.* Minimum Reporting Standards for in vivo Magnetic Resonance Spectroscopy (MRSinMRS): Experts’ consensus recommendations. *NMR Biomed* **34**, e4484 (2021).
