# Supplementary material for: Cortical glutamate and GABA are related to compulsive behaviour in individuals with obsessive compulsive disorder and healthy controls
Source: Nat Commun. 2023 Jun 27;14:3324. doi: 10.1038/s41467-023-38695-z (PMC10300066; doi:10.1038/s41467-023-38695-z)
Supplement: Supplementary file 3 — Reporting Summary [file 41467_2023_38695_MOESM3_ESM.pdf]

nature portfolio

Corresponding author(s): Professor Trevor W Robbins; Dr Marjan Biria

Last updated by author(s): Apr 2, 2023

Reporting Summary

Nature Portfolio wishes to improve the reproducibility of the work that we publish. This form provides structure for consistency and transparency in reporting. For further information on Nature Portfolio policies, see our Editorial Policies and the Editorial Policy Checklist.

Statistics

For all statistical analyses, confirm that the following items are present in the figure legend, table legend, main text, or Methods section.

n/a	Confirmed	
☐	☒	The exact sample size (n) for each experimental group/condition, given as a discrete number and unit of measurement
☐	☒	A statement on whether measurements were taken from distinct samples or whether the same sample was measured repeatedly
☐	☒	The statistical test(s) used AND whether they are one- or two-sided<br>*Only common tests should be described solely by name; describe more complex techniques in the Methods section.*
☐	☒	A description of all covariates tested
☐	☒	A description of any assumptions or corrections, such as tests of normality and adjustment for multiple comparisons
☐	☒	A full description of the statistical parameters including central tendency (e.g. means) or other basic estimates (e.g. regression coefficient) AND variation (e.g. standard deviation) or associated estimates of uncertainty (e.g. confidence intervals)
☐	☒	For null hypothesis testing, the test statistic (e.g. F, t, r) with confidence intervals, effect sizes, degrees of freedom and P value noted<br>*Give P values as exact values whenever suitable.*
☒	☐	For Bayesian analysis, information on the choice of priors and Markov chain Monte Carlo settings
☒	☐	For hierarchical and complex designs, identification of the appropriate level for tests and full reporting of outcomes
☐	☒	Estimates of effect sizes (e.g. Cohen's d, Pearson's r), indicating how they were calculated

Our web collection on statistics for biologists contains articles on many of the points above.

Software and code

Policy information about availability of computer code

Data collection	No code was produced by the authors to collect data in this study. To inquire about the code used for the contingency degradation task please contact the developer of the task, Professor Karen Ersche (ke220@medschl.cam.ac.uk).
Data analysis	The open source software MRspa (version v1.5f) and LCModel (version 6.2-3) were used for pre-processing and model fitting of the spectra. The SPM12 was used to pre-process and segment the structural MP2RAGE images. A MATLAB (version R2018-a) script was made to extract the gray matter, white matter and CSF within each individual voxel, for each subject using their segmented images. Python version 3.7.6 was used to perform the statistical data analysis (t-test, Pearson or Spearman rank correlation coefficients, mean and SD), SPSS version 28 (SPSS IBM) was used for the mixed ANOVA of the behavioral data as this analysis option is not yet available in Python. Rstudio Version 3.0 was used for the Hierarchical Linear Modeling. MATLAB R2018a was used for the tissue correction of the spectroscopy data. Lastly, the p-values were corrected for False Discovery Rates (FDR) according to Benjamini & Hochberg (1995) for each section of the results, with $p < 0.05$. The source code used for the FDR calculation can be found on GitHub: https://github.com/carbocation/falsediscovery.

For manuscripts utilizing custom algorithms or software that are central to the research but not yet described in published literature, software must be made available to editors and reviewers. We strongly encourage code deposition in a community repository (e.g. GitHub). See the Nature Portfolio guidelines for submitting code & software for further information.

Data

Policy information about availability of data

All manuscripts must include a data availability statement. This statement should provide the following information, where applicable:

- Accession codes, unique identifiers, or web links for publicly available datasets
- A description of any restrictions on data availability
- For clinical datasets or third party data, please ensure that the statement adheres to our policy

The data generated in this study are provided in the Source Data and Supplementary Data files which can be accessed here: https://doi.org/10.5281/zenodo.7737126. The raw MRS data will be provided upon request and with restricted access. The latter is due to the inability to fully anonymize the neuroimaging data.

Human research participants

Policy information about studies involving human research participants and Sex and Gender in Research.

Reporting on sex and gender	This study included 30 healthy volunteers (15 female, 15 male) and 31 patients with OCD (17 female, 14 male). Besides one patient (with the male sex and the gender non-binary), the sex and gender of all participants were the same. As the groups included similar numbers of male versus female sex, and the gender and sex were the same in most participants, we did not need to control for them in our analysis. Additionally, we did not have any hypotheses about the impact of gender or sex on neurometabolites in OCD.
Population characteristics	The groups were matched for age, sex and gender. See above.
Recruitment	Healthy individuals were recruited from the community, were all in good health, unmedicated and had no history of neurological or psychiatric conditions. Participants with OCD were recruited through an approved advertisement on the OCD action website (www.ocdaction.org.uk) and local support groups and via clinicians in East Anglia. All OCD participants were screened by a qualified psychiatrist of our team (A.S.) to confirm a primary OCD diagnosis. Additionally, the Mini International Neuropsychiatric Inventory (MINI) was used to confirm the absence of any co-morbid psychiatric conditions. All participants received monetary compensation for taking part in this study.<br><br>A potential self-selection bias could arise from the fact that the more severe OCD cases could not take part in this study due to the nature of their symptoms and the COVID-19 pandemic. As a result, the lack of group differences within the supplementary motor area and the occipital lobes may have been due to this self-selection bias, rather than a true absence of differences.
Ethics oversight	This study was approved by the East of England - Cambridge South Research Ethics Committee (REC 16/EE/0465).

Note that full information on the approval of the study protocol must also be provided in the manuscript.

Field-specific reporting

Please select the one below that is the best fit for your research. If you are not sure, read the appropriate sections before making your selection.

☐ Life sciences ☒ Behavioural & social sciences ☐ Ecological, evolutionary & environmental sciences

For a reference copy of the document with all sections, see nature.com/documents/nr-reporting-summary-flat.pdf

Behavioural & social sciences study design

All studies must disclose on these points even when the disclosure is negative.

Study description	This study consists of a neuroimaging and a behavioral section (taking place outside the scanner). The neuroimaging part has a cross-sectional design comparing the two groups, whereas, the behavioral part has a mixed-design (within subjects, between conditions, and between groups).
Research sample	Healthy volunteers were recruited from the community (N = 30; 15 female; aged 32.7 in average +/- 6.4 years), and patients with OCD (N = 31; 17 female, aged 31 in average +/- 4.5 years) were recruited through an approved advertisement on the OCD action website (www.ocdaction.org.uk) and local support groups and via clinicians in East Anglia.<br><br>The sample may not be representative of the whole sample of the OCD population as the more severe, and the milder cases were not included in this study due to the limitations caused by the pandemic and the nature of their disorder. The milder cases were deliberately excluded to make sure the group differences are due to a clear OCD diagnosis and not OCD symptoms.
Sampling strategy	Healthy volunteers were included in the study if their Obsessive and Compulsive Inventory (OCI; Foa et al., 1998) score was below the

	threshold of 42, and if they had no previous psychiatric condition, and were not being treated for any psychiatric or mental health condition. OCD patients with a primary diagnosis other than OCD were not included in the study. Additionally, patients with comorbidities such as depressive or anxiety symptoms, that were not a consequence of their OCD, were not included in the study. General exclusion criteria for both groups were substance dependence, neurological or medical illnesses or head injury, and not passing the MRI safety criteria (e.g. any metal above the shoulders, shiny tattoos, asthma, etc). All participants had normal or corrected- to normal vision and hearing. As there were no previous 7T studies including OCD patients, 1.5T and 3T studies of the NAA were used to estimate the desired sample size for our study. A power of 80% could be achieved by a sample of 39 using 1.5T scanners (Steen et al., 2005), and it comes down to 29 subjects per group using a 3T scanner (Yücel et al., 2007). We chose the sample size of 30, although we hypothesized that a smaller sample size would give us a power of 80%, considering our scanner was more powerful (7-Tesla).
Data collection	For the behavioral data collection, conducted outside the scanner, participants were seated in front of a 13 inch touch screen SAMSUNG laptop in a comfortable viewing distance with the experimenter always present. They filled out the questionnaires used in this study before arriving to the testing session using an online link sent to them (except for the STAI-state that they had to fill out with pen and paper on the day of testing and in person).<br><br>For the neuroimaging part, single-voxel proton MRS scans were collected using a 7T Siemens Magnetom-Terra scanner. The scanner was equipped with a Nova single-channel transmit, and 32-channel array head coil for signal reception (Nova Medical). During the scan, the researcher was in the scanner's control room with a trained radiographer. The researcher (M.B.) placed the voxels manually, while the trained radiographer performed the rest of the scanning protocol.<br><br>The radiographers collecting the MRS data and the researcher collecting the behavioral data were blind to all study hypotheses (e.g. group differences in neurometabolites, and increased habitual responding in patients) .
Timing	The study started from September 2020 and ended by July 2022.
Data exclusions	At the preprocessing step of the MRS data using MRspa, individual average files that were corrupted were removed. In the OCD group the following averages were removed: within the OCC [1, 4, and 1] averages and within the SMA [2, 2, and 3] averages were removed for 3 subjects. In the HV group, only one subject had a single corrupted average file out of 64 for the OCC voxel. For another healthy participant, due to a data collection error, 54 averages were collected instead of 64.<br><br>In order to avoid exclusion of values that are disorder/group specific and can provide insight into the nature of the OCD, a straight cut-off score (which is usually used) was not used in this study. Instead, per metabolite and per group, the average and standard deviations were calculated for Cramér-Rao Lower Bound of each metabolite and per individual metabolite concentration. Values larger than 2SD from the mean of each group were excluded for both measures. According to this criteria, the following data were excluded: within the SMA voxel, GABA in 1 healthy and 1 OCD subjects, and Gln in 2 OCD patients, within the OCC voxel, 2 Glu and Gln in 2 healthy subjects, and 1 GABA in a healthy subject, and 3 GABA in patients. One ACC and one Occipital lobe voxel were excluded for one OCD patient due to error during data collection.
Non-participation	Two OCD patients dropped out of the study before doing the scan, due to the anxiety caused by their obsessive thoughts.
Randomization	Participants were not randomly allocated to groups as our hypothesis was based on whether or not someone has an OCD diagnosis. However, we did aim to match the groups for sex, age and IQ (~education level).

Reporting for specific materials, systems and methods

We require information from authors about some types of materials, experimental systems and methods used in many studies. Here, indicate whether each material, system or method listed is relevant to your study. If you are not sure if a list item applies to your research, read the appropriate section before selecting a response.

Materials & experimental systems

n/a	Involved in the study
☒	☐ Antibodies
☒	☐ Eukaryotic cell lines
☒	☐ Palaeontology and archaeology
☒	☐ Animals and other organisms
☒	☐ Clinical data
☒	☐ Dual use research of concern

Methods

n/a	Involved in the study
☒	☐ ChIP-seq
☒	☐ Flow cytometry
☐	☒ MRI-based neuroimaging

Magnetic resonance imaging

Experimental design

Design type	Magnetic Resonance Spectroscopy and Magnetic Resonance Imaging at rest (eyes open)
Design specifications	All subjects performed one scan, lasting about 70 mins. After performing a whole-brain T1-weighted MP2RAGE images (~ 10 mins), 64 spectra were collected for each of the 3 voxels of interest (~ 20 mins).

Behavioral performance measures

This scan was not task based. However, the Habitual versus goal-directed behavior was measured outside the scanner, using a contingency degradation task used by Ersche et al. (2021), which consisted of 8 blocks of 120 trials, lasting 1 second each.

Participants were presented with a white vase on the screen which could be filled with flowers when the space bar key was pressed. A reward of £0.20 with different probabilities, could be gained by either performing an action (~ pressing the space bar) or without any action, depending on the condition. There were 3 different conditions each with different probabilities for winning money without performing any action. Blocks 1,2,3 and 6 were the non-degraded condition, with a probability of 0.6 to win £0.20 after pressing the space bar and 0.0 to win the money without any action. Blocks 4 and 7 formed the partially degraded condition with the probabilities of 0.6 and 0.3 to win the money with and without any action, respectively. Lastly, blocks 5 and 8 formed the third and fully-degraded condition, with the probabilities of 0.6 and 0.6 to win the money either with or without any action, respectively.

To study habitual control, an habitual responding index was created by subtracting the responses during the non-degraded condition (probability of 0.60) from the fully degraded condition (probability of 0.30 or 0) with a lower probability of gaining rewards.

Acquisition

Imaging type(s)

Structural (MP2RAGE) MRI + Magnetic Resonance Spectroscopy (MRS)

Field strength

7 Tesla

Sequence & imaging parameters

The following specifications were used to acquire the MP2RAGE structural images: echo time = 4300 ms, repetition time = 1.99 ms, inversion times (1/2)= 840/2370 ms, flip angles = 5/6°, acceleration factor (A ≫ P) = 3, bandwidth = 250 Hz/px, voxel size = 0.75 mm.

For the single-voxel proton Magnetic Resonance Spectroscopy, a short-echo semi-LASER sequence was used to acquire the spectra, collecting 64 repetitions and time/echo time of 5000/26 ms. For each voxel, the FASTESTMAP sequence for shimming, and variable radio frequency pulses with optimised relaxation delay (VAPOR) for water suppression calibration were used.

Area of acquisition

Three voxels were placed in 1) Anterior Cingulate Cortex or ACC (consisting of Brodmann areas 24 and 32), 2) Supplementary Motor Area or SMA (Brodmann area 6; both SMA and pre-SMA were included in this voxel), and 3) Occipital Cortex as a control region. The exact locations of the voxels within the ACC and SMA were selected based on the existing OCD literature.

Diffusion MRI ☐ Used ☒ Not used

Preprocessing

Preprocessing software

SPM12 was used to preprocess the structural data, whereas the MRspa and LCModel (version 6.2-3) were used to preprocess the spectroscopy data.

Normalization

The structural data were normalized using the SPM12 template to produce native-space segmented images. Creatine and phosphocreatine were used to reference the spectroscopy data (this is a default step by LCModel).

Normalization template

See above.

Noise and artifact removal

In addition to the FASTESTMAP sequence for shimming, and variable radio frequency pulses with optimised relaxation delay (VAPOR) for water suppression calibration, metabolites outside 2SD from the mean were removed for both concentration levels and Cramér-Rao Lower Bound of individual metabolites. This was performed separately per groups to take into account the abnormal and yet group specific values.

Moreover, a segmentation analysis was performed using SPM12 and the MP2RAGE images to extract tissue fractions for each subject for Gray Matter and White Matter, and Cerebrospinal fluid, and performed partial volume corrections within subjects according to Harris et al. (2015) for GABA, and Provencher (2021) for the rest of the metabolites to make sure the results are not due to differences in brain structure.

Volume censoring

For the preprocessing step with the MRspa, the same researcher (MB) has paged through each transient/individual average file (64 in total) and dropped any that look corrupted.

We are not aware of an equivalent method in the MRS world to perform the scrubbing that is done in fMRI with certain measured indices such as the framewise/absolute displacement or DVARS.

Statistical modeling & inference

Model type and settings

The spectra were generated by the software LCModel (version 6.2-3), using a nearly model-free constrained regularization method. Approximate maximum-likelihood estimates of all metabolites and their uncertainties as measured by Cramér-Rao Lower Bound were obtained. The analysis of metabolite concentrations with LCModel is automated and based on a fit of the smoothest line shape and baseline to the raw data (Provencher, 2001). The LCModel setting was set to include phase, frequency and Eddy-Current corrections, and a CSF fraction of zero.

Effect(s) tested	One-sided independent sample t-tests were used to study the difference in the neurometabolites between the two groups (and the clinical measures). Whereas, to test the difference in quality measure variables such as the CRLB, FWHM, and brain tissues, a two-sided sample t-test was used. For the behavioral data, a mixed repeated measure ANOVA was employed to study the within and between group effects. Additionally, the relationship between metabolites and the behavioural index (explained above) was studied using either Pearson or Spearman Rank correlation coefficient in case of normally and non-normally distributed data, respectively.

Specify type of analysis: ☐ Whole brain ☒ ROI-based ☐ Both

Anatomical location(s)	The voxels were placed manually by the same researcher (M.B.), and chosen based on the x,y,z coordinates of reported findings in patients with OCD within the anterior cingulate cortex and supplementary motor area. The size of the voxels were selected after a pilot study (27 scans) to decide the best size to increase the signal to noise ratio and yet include the maximum amount of gray matter within these Brodmann areas (24 and 32 for ACC, and 6 for SMA and pre-SMA).
Statistic type for inference<br>(See Eklund et al. 2016)	The article by Eklund et al is not relevant for our study. The statistics type for inference (Eklund et al, 2016) is directed to fMRI analysis, and to the voxelwise and cluster inference. We performed MRS in this study and so the statistical constraints are much less severe. Since to collect fMRI data, thousands of voxels are compared against each other, so there is a high chance that some voxels will appear significant as false positives. However, in the context of our study, we only have three voxels in the brain (SMA, ACC and OCC) and are looking at 6 metabolite ratios within each voxel, for which we perform an FDR correction.
Correction	P-values were corrected for False Discovery Rates (FDR) according to the correction suggested by Benjamini & Hochberg (1995) for each section of the results, with $p < 0.05$. The source code used for the FDR calculation can be found on GitHub: https://github.com/carbocation/falsediscovery.

Models & analysis

n/a	Involved in the study
☒	☐ Functional and/or effective connectivity
☒	☐ Graph analysis
☒	☐ Multivariate modeling or predictive analysis
