# Supplementary material for: Cortical glutamate and GABA are related to compulsive behaviour in individuals with obsessive compulsive disorder and healthy controls
Source: Nat Commun. 2023 Jun 27;14:3324. doi: 10.1038/s41467-023-38695-z (PMC10300066; doi:10.1038/s41467-023-38695-z)
Supplement: Supplementary file 2 — Peer Review File [file 41467_2023_38695_MOESM2_ESM.pdf]

nature portfolio

Peer Review File

Cortical glutamate and GABA are related to compulsive behaviour in individuals with obsessive compulsive disorder and healthy controls

Open Access This file is licensed under a Creative Commons Attribution 4.0 International License, which permits use, sharing, adaptation, distribution and reproduction in any medium or format, as long as you give appropriate credit to the original author(s) and the source, provide a link to the Creative Commons license, and indicate if changes were made. In the cases where the authors are anonymous, such as is the case for the reports of anonymous peer reviewers, author attribution should be to 'Anonymous Referee' followed by a clear attribution to the source work. The images or other third party material in this file are included in the article's Creative Commons license, unless indicated otherwise in a credit line to the material. If material is not included in the article's Creative Commons license and your intended use is not permitted by statutory regulation or exceeds the permitted use, you will need to obtain permission directly from the copyright holder. To view a copy of this license, visit http://creativecommons.org/licenses/by/4.0/.

Reviewer #1 (Remarks to the Author):

Summary:
The manuscript describes ultra-high field (7T) 1H-MRS research with the purpose detecting neurometabolite level differences in two frontal areas (SMA and ACC) important for obsessive compulsive disorder (OCD) and compulsive behavior in general. The authors investigated differences in baseline levels of the most prominent excitatory and inhibitory neurometabolites between a group of OCD patients and healthy controls (HC) (glutamate and gamma-aminobutyric acid (GABA)), as well as regional glutamate:GABA ratios. In addition, the authors tried to link glutamate, GABA and glutamate:GABA ratios to clinical outcomes related to compulsivity as well as task outcomes reflecting habitual control in OCD and HC. By the use of ultra-high field 1H-MRS, the authors address the important limitation of spectral resolution that is often seen for 1H-MRS at lower field strength and are able to assess glutamate and glutamine separately. This is therefore one of the strengths of the current manuscript. Other strengths include a clear structure and concise way of writing as well as linking the findings to both clinical and behavioural aspects of compulsivity. However, it should be mentioned that the argumentation for clinical cut-offs, the reliability of assessing GABA with the used sequence, and explanation for metabolite but not behavioural (index) differences feels a bit thin and should be addressed.

Major concerns:

- In the second paragraph of the introduction (line 91), the authors claim that their 7T semi-LASER sequence is optimized for reliable (and separate) quantification of glutamate, glutamine and GABA. Although this might be through for glutamate and glutamine, reliably measuring GABA often requires a specialized sequence (MEGA-sLASER) or additional editing of the spectra. Although the quality measures (GABA CRLBs) provided in the supplement indicate that the GABA could be interpreted, a word of caution or a reference showing that GABA can be reliably quantified with semi-LASER sequence can strengthen the manuscript. This notion or additional argumentation is especially important for the findings related to E:I ratios.

- In the second paragraph of the methods, the clinical cut-off for the YBOCS is set at 12. The reference provided argues in favor for the use of a YBOCS cut-off of 12 to test the effectiveness of therapy in clinical trials, however the current manuscript describes a cross sectional study. I would like to ask the authors why this threshold was chosen and perhaps state that.

- In line with the previous point; in paragraph seven of the discussion (line 18), it is said that HC OCI scores are likely to be not normally distributed as a result of excluding HC subjects with OCI scores of >42 (a score which serves as the clinical threshold, although is not diagnostic). How many HC actually surpass that OCI threshold? And are they excluded from the other analyses (baseline metabolite comparisons as well as E:I ratio comparisons for the SMA and ACC)? This would, in my opinion be preferable, since it is hard to argue that these high scoring HC are representative for the group they are in.

- The authors decided to use the occipital cortex as control region (in contrast to the more OCD-specific ACC and SMA). However, multiple studies in OCD showed that the occipital region is not an irrelevant brain region for OCD, showing aberrant function in for instance emotional processing. In the results, the authors also show that the glutamate-GABA relationship that is found in controls in all 3 regions (ACC, SMA, OCC) is aberrant in OCD in both SMA and OCC, which is not unexpected based on the recent literature on the role of the occipital cortex in deviant emotional and cognitive processing in OCD. Same concerns for the aberrant Glu levels in the OCC. I think that in the discussion section of the manuscript, the occipital findings need more elaboration and citation of the relevant literature.

Minor concerns:
- In the second paragraph of the introduction (line 99), the authors explain their second hypothesis regarding the proposed bias of OCD patients towards bias towards habitual control rather than being goal-directed. This comes out of the blue as the rest of the paragraph explains metabolites and brain regions related to OCD. This could be addressed by adding a more fluent transition.

- In the second paragraph of the methods, the inclusion and exclusion criteria for both HC and OCD are described. According to the manuscript, HC are not allowed to have psychiatric conditions. For patients it is stated that the MINI is used, but for HC this is not stated. Please add.

- In the paragraph three of the discussion it is mentioned that the observed results are independent of structural change in gray and white matter. A partial volume correction is used to correct the metabolite levels for differences in voxel grey and white matter composition (as a result of voxel placement). It does however not provide substantial evidence about potential structural alterations between patients and HC. To make this claim voxel based morphometry would be the preferred method. Please make the structural part of that sentence better suited to the method it is tested against. NAA as a marker for neuronal integrity seems more suited to address this.

- In the results section (line 223) it is mentioned that OCD patients do perform similar on the task as HC (and figure S3 shows that too). You would expect a task that assessed the balance between habitual and goal-directed control to show different outcomes for groups with high vs low compulsivity. Interestingly, despite the absence of task differences, ACC glutamate:GABA ratio and the behavioral index do correlate for patients only. This is an interesting finding, but potential reasons are not discussed in the discussion (i.e. ACC involvement serves compensatory purpose). It would be interesting to see some additional argumentation on this point (both regarding the lack of behavioural effect of group and regarding the altered relation with Glu:gaba ratio in absence of behavioral deficit).

- In table S1 the CRLB of NAA (M +- SD) are both 1.0 + - 0.0, is this correct?

- Instead of figure S1 (with demographic and clinical characteristics), I would prefer a table with mean (+/- SD) and statistics, since I think it is clearer. I also think that descriptives table should be part of main manuscript (instead of supplements).

Reviewer #2 (Remarks to the Author):

This manuscript details a study of glutamate and GABA in subjects with OCD. The authors showed that glutamate levels in the SMA were related to clinical measures of OCD and that measures of habitual control correlated with the ratio of glutmate:GABA, which is commonly used as a measure of E-I imbalance.

The work appears to be of high quality, and there aren't any issues with the data acquisition and subsequent analysis of the data. However, there are a few places where there's some confusion or where it would be good to have more detailed descriptions. As it stands, some parts of the methods don't provide sufficient detail to reproduce the study. I think the manuscript is worthy of publication, subjects to a few modifications.

One positive aspect of the study is that the authors used semi-LASER rather than the commonly used PRESS localisation method. This will provide improved spatial specificity for the metabolites that were quantified.

The authors make claim to several firsts. In the introduction, they say there are no rigorous studies of glutamate in humans. This is not true, as can be shown by a quick Pubmed search. In the discussion, they also claim to be first to show relationships

between clinical scores and neurochemical indices. Again, this is not the case. Zheng et al showed similar comparisons in 2020.

The results in table 1 should have the same number of digits after the decimal point for the means and SD. For the OCD group, please give the additional figures for Glx in the ACC and Glu/GABA in the SMA, both for the OCD group. These are currently listed as 16±1.13 & 4±1.09 respectively.

The number of non-water-suppressed acquisitions should be given in the acquisition section of the methods rather than (or as well as) in the data preparation and analysis section. Additionally, it looks like the TR and TE have been swapped for the MPRAGE acquisition?

The MRS analysis section is confusing. When the authors say they used LC model to generate "model spectra", what do they mean? Are they just referring to the fit? It's unusual to use LC model to generate model spectra, which is typically synonymous with the basis set. It's stated that a simulated basis set was used. If this was simulated by the authors, details should be provided. For example, what software was used? Were real RF pulse shapes and timings used? Was the simulation spatially averaged? If the basis set was provided by another source, details and references should be given.

The type of referencing for the absolute scaling of the metabolite concentrations is confusing. On line 487, it's stated that concentrations were relative to Cr & PCr. The next sentence says that water referencing was used.

The manuscript states that Ashley Harris' method was used for correcting the GABA concentrations for tissue composition. Since that paper was a 3 T study, where did the authors obtain their T1 & T2 values for applying the relaxation corrections? Were they measured, or were literature values used?

A recent set of experts' consensus papers have recently outlined minimum reporting standards for MRS studies. The authors should consider including the checklist in their supplementary materials.

Dr David J Lythgoe
Department of Neuroimaging
King's College London

Reviewer #3 (Remarks to the Author):

This manuscript describes MRS measures at 7T of glutamate and GABA in ACC, SMA, and OCC together with behavioral assessment in a contingency degradation task in 31 subjects with OCD and 30 matched controls. Strengths of this work include an adequate N, imaging at 7T, and inclusion of a theory-driven behavioral task. Correlational analyses within groups are strong, and the relationships shown between ACC glutamate in OCD and a behavioral measure of compulsivity are novel and interesting. Comparisons between groups are less consistently robust. There are several additional points that require further consideration in a revision.

Introduction.

The review of past literature on glutamate dysregulation in OCD is startlingly sparse, consisting of citation of two book chapters. Past MRS studies are inconclusive but should nevertheless be acknowledged, perhaps by citing the Brennan et al 2013. The cleanest demonstrations of glutamate dysregulation in OCD come from a pair of CSF studies by Chakrabarty and colleagues that should be acknowledged. The 2012 MRS study of GABA

in the ACC by Simpson and colleagues should likewise be noted.

Results.

SNR in the ACC is significantly better in OCD than in controls. What do the authors make of this? Could it lead to a greater ability to detect significant relationships in OCD than in controls (as in Fig 3C)?

The weakening or loss of correlation between measured Glu and GABA levels in SMA and OCC in OCD is interesting but is not clearly demonstrated by the simple correlations presented. It is plausible that the effects claimed here are the result of increased noise in the OCD group, rather than a loss of the correlation, or even that they represent a Type 2 error. If the authors wish to make a claim about difference between groups they need to perform an analysis that directly compares groups, such as a multi-level regression. If such an analysis does not show statistically significant effects of diagnosis, then the claims made here need to be moderated.

Significance values in Table 1 should be FDR corrected (as are the p values presented in Figure 1). In this table it is stated that outliers were excluded; the exclusions are listed in the Methods but should be listed in the table to facilitate data interpretation. (There seem to be quite a lot of exclusions, though they are not obviously biased towards one group of the other.)

Most patients were medicated; this lack of matching with controls is an acknowledged limitation of this study. Were any efforts made to stratify analysis by medication status? (This may be impossible to do rigorously because of the small number of unmedicated subjects.)

Discussion

It is stated that these are the first measurements of glutamate and GABA in the ACC in OCD. This statement is not accurate. Simpson et al 2012 measured GABA. Brennan et al 2015, among others, reported on glutamate. There is, plausibly, benefit to doing these measurements at 7T, but if the authors wish to claim that all previous reports on these mattesr are invalid they need to make a strong argument to that effect, not just ignore relevant past work.

It is stated that the reported results are 'independent of gray and white matter and CSF changes as well as changes in NAA, as a measure of neural integrity'. I infer that the reference to independence from gray and white matter and CSF changes refers to the partial volume correction that was applied to the data; this is fair enough but should be stated more clearly. I do not see where the authors have controlled for NAA in their analysis of Glu and GABA; if they want to make this claim they should probably include NAA as a covariate.

Insensitivity to contingency degradation previously reported by this group in OCD (ref 38) does not replicate in this study, using the same task. This should be commented upon in more detail – the authors note that ref 38 used a 'more parametric and detailed' paradigm, but it is hard to see how this is adequate to explain the complete lack of any even trend-level effects of diagnosis in the current data.

Response to Reviewer 1

Summary:
The manuscript describes ultra-high field (7T) 1H-MRS research with the purpose detecting neurometabolite level differences in two frontal areas (SMA and ACC) important for obsessive compulsive disorder (OCD) and compulsive behavior in general. The authors investigated differences in baseline levels of the most prominent excitatory and inhibitory neurometabolites between a group of OCD patients and healthy controls (HC) (glutamate and gamma-aminobutyric acid (GABA)), as well as regional glutamate:GABA ratios. In addition, the authors tried to link glutamate, GABA and glutamate:GABA ratios to clinical outcomes related to compulsivity as well as task outcomes reflecting habitual control in OCD and HC. By the use of ultra-high field 1H-MRS, the authors address the important limitation of spectral resolution that is often seen for 1H-MRS at lower field strength and are able to assess glutamate and glutamine separately. This is therefore one of the strengths of the current manuscript. Other strengths include a clear structure and concise way of writing as well as linking the findings to both clinical and behavioural aspects of compulsivity. However, it should be mentioned that the argumentation for clinical cut-offs, the reliability of assessing GABA with the used sequence, and explanation for metabolite but not behavioural (index) differences feels a bit thin and should be addressed.

Major concerns:

1) In the second paragraph of the introduction (line 91), the authors claim that their 7T semi-LASER sequence is optimized for reliable (and separate) quantification of glutamate, glutamine and GABA. Although this might be through for glutamate and glutamine, reliably measuring GABA often requires a specialized sequence (MEGA-sLASER) or additional editing of the spectra. Although the quality measures (GABA CRLBs) provided in the supplement indicate that the GABA could be interpreted, a word of caution or a reference showing that GABA can be reliably quantified with semi-LASER sequence can strengthen the manuscript. This notion or additional argumentation is especially important for the findings related to E:I ratios.

We have now added a word of caution in our methods section (lines 608-611) about MEGA-sLASER sequence being a specialised sequence to measure GABA and a reference by Hong et al., (2019) measuring GABA within the same scan session using both semi-LASER and MEGA-sLASER showing a similar accuracy level.

“This semi-LASER approach has recently been shown to have similar accuracy to MEGA-sLASER (an edited MRS sequence) for quantification of GABA in human brain at 7T[94] and we expect similar performance for Glu and Gln.”

To explain our choice of sequence, our goal with this study was to investigate **both** Glutamate and GABA in OCD patients and healthy controls (as the referee clearly understood). Therefore, in order to compare and understand how both metabolites interact, we needed to assess them within the same experiment, using the same sequence which is not possible with an edited sequence because the editing pulses disturb nearby metabolites.

2) In the second paragraph of the methods, the clinical cut-off for the YBOCS is set at 12. The reference provided argues in favor for the use of a YBOCS cut-off of 12 to test the effectiveness of therapy in clinical trials, however the current manuscript describes a cross sectional study. I would like to ask the authors why this threshold was chosen and perhaps state that.

The purpose of the YBOCS is to measure symptom severity in patients diagnosed with OCD (Goodman et al., 1989), thus, healthy participants do not have YBOCS data in this study and this has been stated now more clearly in our methods section (line 535). Considering the latter, YBOCS scores were not used in a cross-sectional design, and were rather measured to compare correlations with symptom severity.

We did indeed based the threshold on the recommendation of the cited reference (Lewin et al., 2011). We are not sure why this should be any different for a cross-sectional compared with treatment study, especially because the scale cannot be used in healthy volunteers as pointed out by Goodman et al., (1989). We felt this wider range was appropriate given the requirements of correlational analysis. In fact, we only had 4 out of 31 patients in this "mild" category. We have reanalysed the data excluding those 4 patients and find similar results. We have not included this additional analysis in the revised manuscript but details are available on request.

3) In line with the previous point; in paragraph seven of the discussion (line 18), it is said that HC OCI scores are likely to be not normally distributed as a result of excluding HC subjects with OCI scores of >42 (a score which serves as the clinical threshold, although is not diagnostic). How many HC actually surpass that OCI threshold? And are they excluded from the other analyses (baseline metabolite comparisons as well as E:I ratio comparisons for the SMA and ACC)? This would, in my opinion be preferable, since it is hard to argue that these high scoring HC are representative for the group they are in.

The referee may be mistaken in their assumption that HC with an OCI score >42 were recruited and excluded from some parts of the analysis. However, we thank them for pointing out this lack of clarity in the manuscript. Healthy volunteers with a score higher than 42 were not recruited in this study at all. The "exclusion" criteria has been replaced by "recruitment" criteria in the manuscript (lines 401, 547) to avoid confusion. Table 3 now shows the mean and SD for OCI of both groups.

4) The authors decided to use the occipital cortex as control region (in contrast to the more OCD specific ACC and SMA). However, multiple studies in OCD showed that the occipital region is not an irrelevant brain region for OCD, showing aberrant function in for instance emotional processing. In the results, the authors also show that the glutamate-GABA relationship that is found in controls in all 3 regions (ACC, SMA, OCC) is aberrant in OCD in both SMA and OCC, which is not unexpected based on the recent literature on the role of the occipital cortex in deviant emotional and cognitive processing in OCD. Same concerns for the aberrant Glu levels in the OCC. I think that in the discussion section of the manuscript, the occipital findings need more elaboration and citation of the relevant literature.

Thank you for pointing this out. We agree that we may have under-emphasised the changes in the occipital cortex given evidence from the literature of possible changes in this region in OCD. We have now extensively researched and have noted in the revised manuscript. We agree also that the term 'control region' is not appropriate and so we have now used "comparison region" as a preferable term (lines 103), as our main interest was the comparison of anterior cortex changes (which were hypothesised in OCD) with posterior cortex.

Our study may be one of the first MRS studies of this region in OCD and so could importantly contribute to this literature. However, this may be tempered by our new analyses of the MRS changes in response to this referee's point 7 (and also referee 3) below, taking into account NAA OCC (or other regions) in OCD, analysis of covariance indicated that the glutamate increase in OCD no longer attained statistical significance. Hence, given also that we found no functional correlate of OCC glutamate, we have thought it appropriate not to highlight this possible change. We have however now added more on OCC in our Discussion (lines 336-341):

"Although our hypothesis of elevated Glu in the ACC of patients with OCD was confirmed, we also found significant increases in the occipital cortex (though not SMA) in this group suggesting that such changes may not be limited to frontal regions in OCD, consistent with evidence from several other neuroimaging studies[45–50]. However, there were no correlations shown for any of the behavioral measures for OCC. Moreover, following corrections based on FDR or NAA levels this finding was no longer significant, hence its functional relevance, if any, is unclear."

Minor concerns:

5) In the second paragraph of the introduction (line 99), the authors explain their second hypothesis regarding the proposed bias of OCD patients towards bias towards habitual control rather than being goal-directed. This comes out of the blue as the rest of the paragraph explains metabolites and brain regions related to OCD. This could be addressed by adding a more fluent transition.

We have now deleted that sentence and reordered the text to provide a more fluent transition with a short, more explanatory paragraph (lines 113-118), as below:

"We further correlated neurochemical levels within these regions with measures of compulsivity, and a behavioral index of habitual control- a contingency degradation task, whereby the association between an action and an outcome is uncoupled or degraded[23]. Since compulsive behavior in OCD has been postulated to emerge from an imbalanced cortico-striatal circuitry favoring the habit system we hypothesized that this might be reflected in neurochemical imbalances in the anterior cingulate and SMA."

6) In the second paragraph of the methods, the inclusion and exclusion criteria for both HC and OCD are described. According to the manuscript, HC are not allowed to have psychiatric conditions. For patients it is stated that the MINI is used, but for HC this is not stated. Please add.

The methods is now written more clearly to state which questionnaires were used for which group for inclusion/exclusion purposes.

7) In the paragraph three of the discussion it is mentioned that the observed results are independent of structural change in gray and white matter. A partial volume correction is used to correct the metabolite levels for differences in voxel grey and white matter composition (as a result of voxel placement). It does however not provide substantial evidence about potential structural alterations between patients and HC. To make this claim voxel based morphometry would be the preferred method. Please make the structural part of that sentence better suited to the method it is tested against. NAA as a marker for neuronal integrity seems more suited to address this.

Thank you for raising this important point. This sentence is now changed to avoid making claims about structural changes which could only have been made had we done a VBM analysis (line 354-358).

"The changes we found in ACC Glu and GABA were shown to be independent of the gray and white matter and CSF composition within each individual voxel for each subject. We additionally controlled for neuronal integrity via NAA changes, this resulting not only in the disappearance of the significant elevation in Glu concentration found in the OCC, but also in more significant depletion of ACC GABA levels in the OCD group."

We did of course correct for gray and white matter and CSF ratios within voxels but we agree that correction using NAA levels is a more suitable approach. In fact we have now used this to perform ANCOVA with NAA as a covariate. Some results such as GABA differences between groups actually became stronger while, as mentioned above, OCC Glu differences became insignificant. The latter findings point towards differences in neuronal integrity within groups, despite no differences in NAA levels found. Table 2 now also shows the results of this analysis which has clarified our findings and further supported the effects shown on GABA, as well as glutamate in the anterior cingulate of OCD patients.

8) In the results section (line 223) it is mentioned that OCD patients do perform similar on the task as HC (and figure S3 shows that too). You would expect a task that assessed the balance between habitual and goal-directed control to show different outcomes for groups with high vs low compulsivity. Interestingly, despite the absence of task differences, ACC glutamate:GABA ratio and the behavioral index do correlate for patients only. This is an interesting finding, but potential reasons are not discussed in the discussion (i.e. ACC involvement serves compensatory purpose). It would be interesting to see some additional argumentation on this point (both regarding the lack of behavioural effect of group and regarding the altered relation with Glu:gaba ratio in absence of behavioral deficit).

It was certainly somewhat surprising not to find group differences in the contingency degradation task which therefore do not support a straightforward hypothesis of goal-directed behavior/habitual control imbalance in OCD- and we have noted this in the Discussion. The original finding of impaired contingency degradation performance was found in the context of a more elaborate study on contingency learning by Vaghi et al, rather than in this simplified version of the task which was designed for functional imaging purposes, and so this result is not a simple replication failure. However, it is apparent that there is nevertheless a significant difference in the neural mediation of habitual performance between the groups, perhaps as the referee notes, because of the recruitment of the anterior cingulate as well as the SMA in OCD patients. We have now elaborated on the possibility that this may reflect functional compensatory processes in OCD in the revised Discussion.

The following has been now added to the ms (lines 406-415 and 457-464):

"*Evidence has been reviewed supporting a previous hypothesis suggesting that compulsion in OCD can result from an imbalance in competing neural systems governing instrumental goal-directed and habitual control of behavior with a bias to the latter*[63]*. Part of this evidence has depended on a test of contingency degradation whereby instrumental actions are uncoupled from their outcomes by weakening their predictive correlation. Thus OCD patients continued to respond in some conditions despite showing normal subjective causal judgement of the contingencies, presumably as a consequence of greater habitual control over performance*[64]*. Consequently, in this study we also included a simplified contingency degradation test modified for use in neuroimaging studies and previously shown to be sensitive to deficits in stimulant drug abusers, as well as being validated by a subjective habit questionnaire*[34]*."*

"with OCD impairments previously shown in the more elaborate test paradigm used earlier[64]. This means that no behavioral support was found for the hypothesis that OCD patients exhibit weaker goal-directed behavior or strengthened habitual responding in this test procedure, simplified for the purpose of neuroimaging. Nevertheless, the neural mediation of habitual control in OCD additionally implicated the ACC, in contrast to HVs (Fig. 3c). The ACC may have been recruited in OCD because of its known role in the mediation of action-outcome learning and error prediction, especially under uncertainty[16,17,19,76]. This recruitment possibly reflects a functional compensation in the OCD patients, especially given the classical role of the ACC in response to error-monitoring performance[77–79].This possibility of functional compensation in OCD is consistent with previous evidence from both MRS[40] and error-related negativity studies[80,81]."

9) In table S1 the CRLB of NAA (M +- SD) are both 1.0 + - 0.0, is this correct?
Yes, these values are correct. Since all CRLB for NAA were 1 (as NAA is the easiest peak to measure), the standard deviation was also 0.

10) Instead of figure S1 (with demographic and clinical characteristics), I would prefer a table with mean (+/- SD) and statistics, since I think it is clearer. I also think that descriptives table should be part of main manuscript (instead of supplements).
Figure S1 has been replaced with Table 3 and moved to the main text instead of the Supplementary Information.

Response to Reviewer 2

Summary:

This manuscript details a study of glutamate and GABA in subjects with OCD. The authors showed that glutamate levels in the SMA were related to clinical measures of OCD and that measures of habitual control correlated with the ratio of glutamate:GABA, which is commonly used as a measureof E-I imbalance. The work appears to be of high quality, and there aren't any issues with the data acquisition and subsequent analysis of the data. However, there are a few places where there's some confusion or where it would be good to have more detailed descriptions. As it stands, some parts of the methods don't provide sufficient detail to reproduce the study. I think the manuscript is worthy of publication, subjects to a few modifications. One positive aspect of the study is that the authors used semi-LASER rather than the commonly used PRESS localisation method. This will provide improved spatial specificity for the metabolites that were quantified.

Comments:

11) The authors make claim to several firsts. In the introduction, they say there are no rigorous studies of glutamate in humans. This is not true, as can be shown by a quick Pubmed search. In the discussion, they also claim to be first to show relationships between clinical scores and neurochemical indices. Again, this is not the case. Zheng et al showed similar comparisons in 2020.

On reconsideration of the previous literature it appears that we are reporting for the first time changes in *both* glutamate and GABA in the ACC of OCD patients (and in opposite directions), possibly because of our use of the more powerful 7T scanner (the reduced GABA finding came as a result of our further analysis correcting for NAA levels in addressing the other referees' helpful recommendations). The second set of new findings comprises the significant correlations of neurochemical changes in the ACC and SMA relating to compulsivity. Consequently we have now

clarified our priority claims (to the best of our knowledge) in this revised manuscript to emphasize these novel findings.

We do agree that we may have under-represented the previous MRS literature to some extent, although it is true that previous studies of glutamate have been hindered by a lack of resolution at lower than 7T and have been inconsistent as also supported by the review by Brennan et al., (2013) which we have now also cited in the Introduction. The paper by Zheng et al., (2020) was not cited because the correlation with clinical measures was in fact based on an n of only 9 female patients with diagnosis of skin-picking as well as OCD. Therefore, we did not feel this was worth citing and have not added it to the revision. However, also in response to Referee 3's suggestion, we have now cited in the Introduction and Discussion the important paper by Simpson et al using 3T MRS showing reductions of GABA in the medial prefrontal cortex, although a later paper (also cited) found *increased* levels in the same region, also using 3-T- which supports our general viewpoint of inconsistency. In the Discussion we have now provided a much more detailed survey of the MRS literature for OCD and drawn out the parallels to our present findings as well as the inconsistencies and possible factors causing these.

The following has now been added to the Introduction (lines 82-92) and Discussion (lines 327-335):

"There is considerable evidence of hyperactivity in OCD in certain cortical regions based on blood oxygenation level dependent (BOLD) neuroimaging[4–6] which presumably is a consequence of changes in the excitatory/inhibitory balance in cortical networks resulting from changes in glutamate (Glu) and γ-amino butyric acid (GABA) neurotransmission[9]. Abnormally high levels of Glu within OCD have been suggested in animal models and by human genetic, pharmacological and neurochemical studies[7–9]. However, there have been inconsistent findings using proton magnetic resonance spectroscopy (1H-MRS) to directly measure regional levels of these neurotransmitters (see Brennan et al., 2013[10] and Biria et al., 2021[11] for review). For example, one 3-Tesla (3T) 1H-MRS study found evidence of reduced GABA in the medial prefrontal cortex of OCD patients, suggesting an altered excitatory/inhibitory balance in that region[12]; however a later study found the reverse[13]."

"Previous studies at lower magnet strengths have found evidence for greater Glu concentrations (though less specifically, Glu + Gln measured together as Glx) in this region of the ACC[36,37]. Naaijen et al. (2017) also found increased Glu in the anterior cingulate in children with OCD or autism spectrum disorder using a 3T scanner[38], suggesting that these changes may occur early in life. On the other hand, several studies have found no significant effects in prefrontal cortex (PFC) regions[12,13,39–42] or even reductions[43,44]. For GABA, Simpson et al (2012)[12] also found reductions in a medial PFC voxel including the ACC, and Zhang al (2016)[41] reported a decreasing trend in the ACC and a reduction in the orbitofrontal cortex. However, a more recent study found increased GABA levels in the ACC[13]."

12) The results in table 1 should have the same number of digits after the decimal point for the means and SD. For the OCD group, please give the additional figures for Glx in the ACC and Glu/GABA in the SMA, both for the OCD group. These are currently listed as 16±1.13 & 4±1.09 respectively.

Thank you for pointing this out, two decimal points have now been added similar to the rest of the data points.

13) The number of non-water-suppressed acquisitions should be given in the acquisition section of the methods rather than (or as well as) in the data preparation and analysis section. Additionally, it looks like the TR and TE have been swapped for the MPRAGE acquisition?

Thank you for raising this issue. This has now been corrected to 4300 ms repetition time and 1.99 ms echo time in the acquisition section of the method (line 598).

14) The MRS analysis section is confusing. When the authors say they used LC model to generate "model spectra", what do they mean? Are they just referring to the fit? It's unusual to use LC model to generate model spectra, which is typically synonymous with the basis set. It's stated that a simulated basis set was used. If this was simulated by the authors, details should be provided. For example, what software was used? Were real RF pulse shapes and timings used? Was the simulation spatially averaged? If the basis set was provided by another source, details and references should be given.

We simply meant the fitted components from LCModel. We have altered the manuscript (lines 620-623) to use this terminology:

"LCModel[97] version 6.2-3 was used with an automated fitting routine, to quantify metabolites including GABA, Glu, Gln and NAA, relative to Cr + PCr (creatine plus phosphocreatine). Individual component fitted spectra for those metabolites between 0.5 and 4.2 ppm were extracted for quality inspection."

15) The type of referencing for the absolute scaling of the metabolite concentrations is confusing. On line 487, it's stated that concentrations were relative to Cr & PCr. The next sentence says that water referencing was used.

All of the results reported have been standardised to Cr + PCr signals. We had reported water referencing because it is included in LCModel pipeline. Since we didn't use water referenced results, we have now removed references to the water referencing step from the manuscript and added the following sentence (line 623-624):

"The metabolites were quantified by reference to 8 spectra acquired without water suppression just before or after the 64 spectral repetitions"

16) The manuscript states that Ashley Harris' method was used for correcting the GABA concentrations for tissue composition. Since that paper was a 3 T study, where did the authors obtain their T1 & T2 values for applying the relaxation corrections? Were they measured, or were literature values used?

The corrections made were limited to partial volume corrections, as indicated in the text, using tissue fractions (GM, WM and CSF) obtained by segmenting the structural data within a region of interest corresponding to the voxel acquired. Further corrections based on relaxation were not performed.

17) A recent set of experts' consensus papers have recently outlined minimum reporting standards for MRS studies. The authors should consider including the checklist in their supplementary materials.

Thank you for this suggestion, we have now added a table with the checklist suggested by Lin et al., 2021 in our supplementary material (Table S2) to increase technical standardization of MRS studies.

Response to Reviewer 3

Summary:

This manuscript describes MRS measures at 7T of glutamate and GABA in ACC, SMA, and OCC together with behavioral assessment in a contingency degradation task in 31 subjects with OCD and 30 matched controls. Strengths of this work include an adequate N, imaging at 7T, and inclusion of a theory-driven behavioral task. Correlational analyses within groups are strong, and the relationships shown between ACC glutamate in OCD and a behavioral measure of compulsivity are novel and interesting. Comparisons between groups are less consistently robust. There are several additional points that require further consideration in a revision.

Comments:

Introduction:

18) The review of past literature on glutamate dysregulation in OCD is startlingly sparse, consisting of citation of two book chapters. Past MRS studies are inconclusive but should nevertheless be acknowledged, perhaps by citing the Brennan et al 2013. The cleanest demonstrations of glutamate dysregulation in OCD come from a pair of CSF studies by Chakrabarty and colleagues that should be acknowledged. The 2012 MRS study of GABA in the ACC by Simpson and colleagues should likewise be noted.

Thank you for this point. We agree that we may have under-represented the previous MRS literature to some extent, although it is true that previous studies of glutamate have been hindered by a lack of resolution at lower than 7T and have been inconsistent as also supported by the review by Brennan et al which we have now also cited in the Introduction, together with the CSF studies of Chakrabarty et al. We have now cited in the Introduction and Discussion the important paper by Simpson et al using 3T MRS showing reductions of GABA in the medial prefrontal cortex, although a later paper (also cited) found increased levels in the same region, also using 3-T- which supports our general viewpoint of inconsistency. In the Discussion we have now provided a much more detailed survey of the MRS literature for OCD and drawn out the parallels to our present findings as well as the inconsistencies and possible factors causing these.

The following has now been added to the Introduction (lines 82-92) and Discussion (lines 327-335):

"There is considerable evidence of hyperactivity in OCD in certain cortical regions based on blood oxygenation level dependent (BOLD) neuroimaging[4–6] which presumably is a consequence of changes in the excitatory/inhibitory balance in cortical networks resulting from changes in glutamate (Glu) and γ-amino butyric acid (GABA) neurotransmission[9]. Abnormally high levels of Glu within OCD have been suggested in animal models and by human genetic, pharmacological and neurochemical studies[7–9]. However, there have been inconsistent findings using proton magnetic resonance spectroscopy (1H-MRS) to directly measure regional levels of these neurotransmitters (see Brennan et al., 2013[10] and Biria et al., 2021[11] for review). For example, one 3-Tesla (3T) 1H-MRS study found evidence of reduced GABA in the medial prefrontal cortex of OCD patients, suggesting an altered excitatory/inhibitory balance in that region[12]; however a later study found the reverse[13]."

"Previous studies at lower magnet strengths have found evidence for greater Glu concentrations (though less specifically, Glu + Gln measured together as Glx) in this region of the ACC[36,37]. Naaijen et al. (2017) also found increased Glu in the anterior cingulate in children with OCD or autism spectrum disorder using a 3T scanner[38], suggesting that these changes may occur early in life. On the other hand, several studies have found no significant effects in prefrontal cortex (PFC) regions[12,13,39–42] or even reductions[43,44]. For GABA, Simpson et al (2012)[12] also found reductions in a medial PFC voxel including the ACC, and Zhang al (2016)[41] reported a decreasing trend in the ACC and a reduction in the orbitofrontal cortex. However, a more recent study found increased GABA levels in the ACC[13]."

Results:

19) SNR in the ACC is significantly better in OCD than in controls. What do the authors make of this? Could it lead to a greater ability to detect significant relationships in OCD than in controls (as in Fig 3C)?

We did report in the supplementary material (Table S1) the higher signal levels of Glu, Gln and NAA (but not GABA) in this group. Since the SNR measure is based on the signals of all metabolites present relative to the residual noise level, higher concentrations of key metabolites would seem to be the most plausible explanation for the higher SNR. Despite the increased level of SNR in OCD patients, levels of GABA were in fact significantly lower in that group in the ACC, so despite the higher SNR (measured in the spectrum overall) it is improbable to us that this would provide greater power to detect relationships involving GABA in that group. Therefore, we have not interpreted the higher SNR in the OCD group for the ACC in terms of pathophysiology.

20) The weakening or loss of correlation between measured Glu and GABA levels in SMA and OCC in OCD is interesting but is not clearly demonstrated by the simple correlations presented. It is plausible that the effects claimed here are the result of increased noise in the OCD group, rather than a loss of the correlation, or even that they represent a Type 2 error. If the authors wish to make a claim about difference between groups they need to perform an analysis that directly compares groups, such as a multi-level regression. If such an analysis does not show statistically significant effects of diagnosis, then the claims made here need to be moderated.

Thank you for this helpful point which has encouraged us to perform a more definitive analysis along the lines suggested. Consequently, we have performed an hierarchical linear regression to predict Glu, using GABA levels, while controlling for both groups and voxels. We built 6 models and used an analysis of variance to select the best model which used GABA with an interaction term with Group, and controlled for Voxel positions. This model predicted around 65% of variability in Glu levels and confirmed firstly that Glu levels could be predicted based on GABA concentrations, secondly that the Voxel location was an important source of variance (41%) and thirdly, the Group assignation (HV vs OCD) improved the model by increasing the adjusted-R squared by 2%. The outputs of this model are now presented in Table 1 within the main text and the outputs of the other models are presented in Supplementary Information.

21) Significance values in Table 1 should be FDR corrected (as are the p values presented in Figure 1). In this table it is stated that outliers were excluded; the exclusions are listed in the Methods but should be listed in the table to facilitate data interpretation. (There seem to be quite a lot of exclusions, though they are not obviously biased towards one group of the other.)

Thank you for your suggestion, we have now added a column to Table 1 with the FDR corrected *p*-values. We also mention the exclusions in the table description, in addition to the methods, and the MRS checklist (Table S2, Supplementary Information).

22) Most patients were medicated; this lack of matching with controls is an acknowledged limitation of this study. Were any efforts made to stratify analysis by medication status? (This may be impossible to do rigorously because of the small number of unmedicated subjects.)

Medication has been shown to reduce glutamate (Musazzi et al., 2013), which is opposite to what we found and thus suggests that medication is not responsible for our findings. Unfortunately, only 6 out of our 31 patients were unmedicated so it was not possible to analyse this factor directly. However, the Simpson et al. reductions in GABA in the mPFC (which included the dorsal anterior cingulate region), and increased Glx were both found in unmedicated patients. Moreover, De Salles Andrade et al., (2019) replicated the increase in Glx in unmedicated patients. Therefore, this supportive evidence has now been added to the Discussion (line 463-464):

"Additionally, several of the previous studies showing changes in neurometabolites employed unmedicated OCD samples[9,12,55]"

Discussion:

23) It is stated that these are the first measurements of glutamate and GABA in the ACC in OCD. This statement is not accurate. Simpson et al 2012 measured GABA. Brennan et al 2015, among others, reported on glutamate. There is, plausibly, benefit to doing these measurements at 7T, but if the authors wish to claim that all previous reports on these matters are invalid they need to make a strong argument to that effect, not just ignore relevant past work.

We did not mean to say of course that we are the first to have measured GABA or glutamate in the ACC in OCD! Nor did we mean to imply that previous studies of this are invalid. On careful reconsideration of the previous literature it does however appear that we are reporting for the first time changes in *both* glutamate and GABA in the ACC of OCD patients (and in opposite directions), possibly because of our use of the more powerful 7T scanner which is to our knowledge the first time in OCD patients (the reduced GABA finding came as a result of our further analysis correcting for NAA levels in addressing your helpful recommendation). Consequently we have now clarified our priority claims (to the best of our knowledge) in this revised manuscript, to emphasize these novel findings.

We have now also included references to important studies by Simpson et al., (2012) and important review of Brenan et al., (2013) and included a more comprehensive review of the literature in the discussion. However, taken as a whole their findings have certainly been inconsistent (as also confirmed by the Brennan et al review now cited). Even the elegant Simpson et al study has not been replicated in a recent paper (Li et al., 2019). We have extended our analysis of previous studies in the Discussion to include Brennan et al 2015 among other studies, although we did already cite the paper by Naaijen et al (2017) in children with OCD.

The following has been added to the Discussion (lines 325-335):

"This appears to be the first report of significant changes, in both of these neurometabolites in the same study in adult OCD to date, possibly reflecting the greater sensitivity of 7T ^{1}H-MRS. Previous studies at lower magnet strengths have found evidence for greater Glu (though less specifically, Glu + Gln measured together as Glx) concentrations in this region of the ACC[36,37]. Naaijen et al. (2017) also found increased Glu in the anterior cingulate in children with OCD or autism spectrum

disorder using a 3T scanner[38]*, suggesting that these changes may occur early in life. On the other hand, several studies have found no significant effects in prefrontal cortex (PFC) regions*[12,13,39–42] *or even reductions*[43,44]*. For GABA, Simpson et al (2012)*[12] *also found reductions in a medial PFC voxel including the ACC, and Zhang al (2016)*[41] *reported a decreasing trend in the ACC and a reduction in the orbitofrontal cortex. However, a more recent study found increased GABA levels in the ACC*[13]*."*

24) It is stated that the reported results are 'independent of gray and white matter and CSF changes as well as changes in NAA, as a measure of neural integrity'. I infer that the reference to independence from gray and white matter and CSF changes refers to the partial volume correction that was applied to the data; this is fair enough but should be stated more clearly. I do not see where the authors have controlled for NAA in their analysis of Glu and GABA; if they want to make this claim they should probably include NAA as a covariate.

This is similar to the point raised by Referee 1 above. We have changed this sentence to avoid making claims about structural changes which could only have been made had we done a VBM analysis. We agree that correction using NAA levels is a more suitable approach and in fact have now used this to perform ANCOVA with NAA as a covariate. Some results such as GABA differences between groups actually became stronger while, as mentioned above, OCC Glu differences became insignificant. The latter findings point towards differences in neuronal integrity within groups, despite no differences in NAA levels found. Table 2 now also shows the results of this analysis which has clarified our findings and further supported the effects shown on GABA, as well as glutamate in the anterior cingulate of OCD patients.

The following has now been adapted in the ms (line 354-358).

"The changes we found in ACC Glu and GABA were shown to be independent of the gray and white matter and CSF composition within each individual voxel for each subject. We additionally controlled for neuronal integrity via NAA changes, this resulting not only in the disappearance of the significant elevation in Glu concentration found in the OCC, but also in more significant depletion of ACC GABA levels in the OCD group."

25) Insensitivity to contingency degradation previously reported by this group in OCD (ref 38) does not replicate in this study, using the same task. This should be commented upon in more detail – the authors note that ref 38 used a 'more parametric and detailed' paradigm, but it is hard to see how this is adequate to explain the complete lack of any even trend-level effects of diagnosis in the current data.

It was certainly somewhat surprising not to find group differences in the contingency degradation task which therefore do not support a straightforward hypothesis of goal-directed behavior/habitual control imbalance in OCD- and we have noted this in the Discussion. The original finding of impaired contingency degradation performance was found in the context of a more elaborate study on contingency learning by Vaghi et al, rather than in this simplified version of the task which was designed for functional imaging purposes, and so this result is not a simple replication failure. However, it is apparent that there is nevertheless a significant difference in the neural mediation of habitual performance between the groups, perhaps as Referee 1 noted above, because of the recruitment of the anterior cingulate as well as the SMA in OCD patients. We have now elaborated on the possibility that this may reflect functional compensatory processes in OCD in the revised Discussion.

The following has been now added to the ms (lines 406-415 and 448-458):

"*Evidence has been reviewed supporting a previous hypothesis suggesting that compulsion in OCD can result from an imbalance in competing neural systems governing instrumental goal-directed and habitual control of behavior with a bias to the latter*[63]*. Part of this evidence has depended on a test of contingency degradation whereby instrumental actions are uncoupled from their outcomes by weakening their predictive correlation. Thus OCD patients continued to respond in some conditions despite showing normal subjective causal judgement of the contingencies, presumably as a consequence of greater habitual control over performance*[64]*. Consequently, in this study we also included a simplified contingency degradation test modified for use in neuroimaging studies and previously shown to be sensitive to deficits in stimulant drug abusers, as well as being validated by a subjective habit questionnaire*[34]*.*"

"with OCD impairments previously shown in the more elaborate test paradigm used earlier[64]*. This means that no behavioral support was found for the hypothesis that OCD patients exhibit weaker goal-directed behavior or strengthened habitual responding in this test procedure, simplified for the purpose of neuroimaging. Nevertheless, the neural mediation of habitual control in OCD additionally implicated the ACC, in contrast to HVs (Fig. 3c). The ACC may have been recruited in OCD because of its known role in the mediation of action-outcome learning and error prediction, especially under uncertainty*[16,17,19,76]*. This recruitment possibly reflects a functional compensation in the OCD patients, especially given the classical role of the ACC in response to error-monitoring performance*[77–79]*.This possibility of functional compensation in OCD is consistent with previous evidence from both MRS40 and error-related negativity studies*[80,81]*."*

Reviewer #1 (Remarks to the Author):

I think that the authors did a proper job in revising the manuscript based on the reviewers' comments, including mine. I would like to support publication of this work.

Reviewer #2 (Remarks to the Author):

I am happy with the authors' responses to my original review and I'm happy to recommend publication.

Reviewer #3 (Remarks to the Author):

This manuscript is improved in this revision and is now appropriate for publication, with a few minor additional edits.

I appreciate that the authors have now included a multi-level analysis of the correlations shown in Figure 1. however, by presenting it in detail in the Results but providing no interpretation, they obfuscate the fact that it does not in fact (quite) cleanly support their original claim: that there is a significant difference in the Glu/GABA relationship between diagnoses that depends on region. This claim requires a significant diagnosis x voxel interaction. I have no problem with noting that this term is nearly significant, consistent with the claim (and there is a clearly significant effect of diagnosis, supporting a slightly modified but still interesting claim); but I suggest that the way the model is presented, with much detail and no interpretation, confuses more than it clarifies. I suggest putting much of the description in the Supplement and describing the model more pithily, focusing on the findings: there is a strong effect of voxel, a significant effect of diagnosis, and a trend for the diagnosis x voxel interaction; this provides clear support for the claim that the relationship between glutamate and GABA is altered in OCD, and modest support for the claim that this effect varies by voxel.

A more minor point: in the Introduction, the sentence on lines 109-111 is not in fact a sentence; it is quite unclear what is being said here.

Reviewer 1
I think that the authors did a proper job in revising the manuscript based on the reviewers' comments, including mine. I would like to support publication of this work.

Reviewer 2
I am happy with the authors' responses to my original review and I'm happy to recommend publication.

We thank reviewers 1 and 2 for their positive feedback, and also appreciated their helpful feedback immensely during the previous revision resulting in improvements of our manuscript.

Reviewer 3

This manuscript is improved in this revision and is now appropriate for publication, with a few minor additional edits.

I appreciate that the authors have now included a multi-level analysis of the correlations shown in Figure 1. However, by presenting it in detail in the Results but providing no interpretation, they obfuscate the fact that it does not in fact (quite) cleanly support their original claim: that there is a significant difference in the Glu/GABA relationship between diagnoses that depends on region. This claim requires a significant diagnosis x voxel interaction. I have no problem with noting that this term is nearly significant, consistent with the claim (and there is a clearly significant effect of diagnosis, supporting a slightly modified but still interesting claim); but I suggest that the way the model is presented, with much detail and no interpretation, confuses more than it clarifies. I suggest putting much of the description in the Supplement and describing the model more pithily, focusing on the findings: there is a strong effect of voxel, a significant effect of diagnosis, and a trend for the diagnosis x voxel interaction; this provides clear support for the claim that the relationship between glutamate and GABA is altered in OCD, and modest support for the claim that this effect varies by voxel.

We thank the reviewer 3 for their constructive feedback. The results are now changed by moving most of the description to the Supplementary Information and describe the model more briefly and clearly within the main text.

*"In addition to the correlation analysis above, in order to substantiate the conclusions further, a confirmatory 6 stage hierarchical linear regression model was built to predict Glu levels from GABA concentrations, while controlling for Group (OCD vs HV) and Voxel (OCC, ACC and SMA regions). Analysis of variance was used to select the best model (model4: Glu ~ GABA*Group+Voxel), predicting around 65% of variability in Glu levels ($F(5,167) = 65.10$, $p < 2.2e-16$). Table 1 shows the output summary for this model. There was a significant effect of voxel (ACC, SMA, OCC), a significant effect of group (OCD vs HV), and a trend for the group and voxel interaction. These findings provide a clear support for the claim that the relationship between Glu and GABA is altered in participants with OCD, and only a modest support for the claim that this effect varies by voxel. All model outputs are described in detail in Supplementary Information, including the R code."*

A more minor point: in the Introduction, the sentence on lines 109-111 is not in fact a sentence; it is quite unclear what is being said here.

Thanks for spotting the incomplete sentence, it is now changed to the following:

"The neurocognitive deficits found in the SMA are considered an endophenotype of OCD, possibly related to inefficient neural processing, as measured during a response inhibition task[6]*. The relevance of SMA in OCD is also apparent from symptom improvements following brain stimulation*[26,27]*."*
